# FLRT2 prevents endothelial cell senescence and vascular aging by regulating the ITGB4/mTORC2/p53 signaling pathway

**DOI:** 10.1172/jci.insight.172678

**Published:** 2024-04-08

**Authors:** Hyun Jung Hwang, Donghee Kang, Jae-Ryong Kim, Joon Hyuk Choi, Ji-Kan Ryu, Allison B. Herman, Young-Gyu Ko, Heon Joo Park, Myriam Gorospe, Jae-Seon Lee

**Affiliations:** 1 Research Center for Controlling Intercellular Communication and; 2 Department of Molecular Medicine, College of Medicine, Inha University, Incheon, Korea.; 3 Program in Biomedical Science and Engineering, Inha University, Incheon, Korea.; 4 Department of Biochemistry and Molecular Biology and; 5 Department of Pathology, College of Medicine, Yeungnam University, Daegu, Korea.; 6 Department of Urology, College of Medicine, Inha University, Incheon, Korea.; 7 Laboratory of Genetics and Genomics, National Institute on Aging, Intramural Research Program, NIH, Baltimore, Maryland, USA.; 8 Division of Life Sciences, Korea University, Seoul, Korea.; 9 Department of Microbiology, College of Medicine, Inha University, Incheon, Korea.

**Keywords:** Cell biology, Vascular biology, Cellular senescence

## Abstract

The roles of fibronectin leucine-rich transmembrane protein 2 (FLRT2) in physiological and pathological processes are not well known. Here, we identify a potentially novel function of FLRT2 in preventing endothelial cell senescence and vascular aging. We found that FLRT2 expression was lower in cultured senescent endothelial cells as well as in aged rat and human vascular tissues. FLRT2 mediated endothelial cell senescence via the mTOR complex 2, AKT, and p53 signaling pathway in human endothelial cells. We uncovered that FLRT2 directly associated with integrin subunit beta 4 (ITGB4) and thereby promoted ITGB4 phosphorylation, while inhibition of ITGB4 substantially mitigated the induction of senescence triggered by FLRT2 depletion. Importantly, FLRT2 silencing in mice promoted vascular aging, and overexpression of FLRT2 rescued a premature vascular aging phenotype. Therefore, we propose that FLRT2 could be targeted therapeutically to prevent senescence-associated vascular aging.

## Abstract 2

FLRT2 critically regulates endothelial cell senescence and vascular aging via induction of the ITGB4/AKT/mTORC2/p53/p21 signaling pathway.

## Graphical abstract

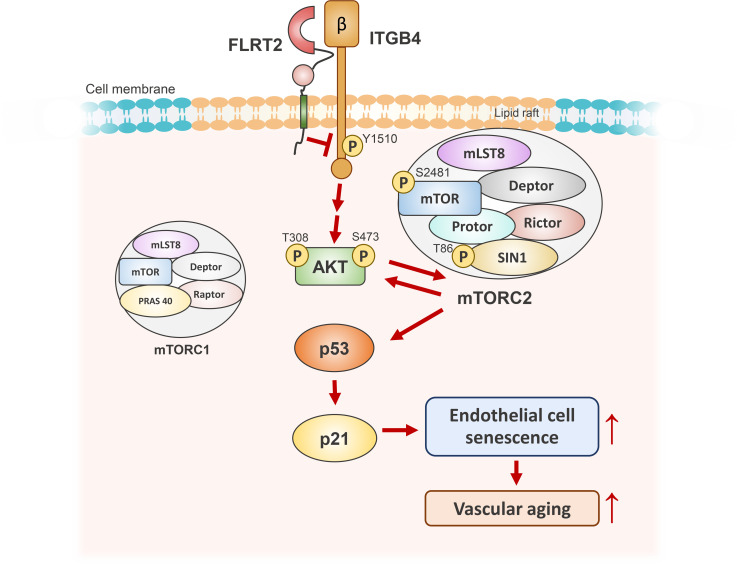

## Introduction

Cellular senescence is characterized by indefinite loss of proliferation potential. Replicative senescence occurs following critical telomere shortening after cells undergo extended rounds of division (1, 2). Premature senescence is triggered by a range of acute intrinsic and extrinsic signals, such as strong mitotic stimuli, oxidative stress, and DNA damage (3, 4). Major signaling pathways driving cellular senescence are the p53/p21 and the RB/p16 tumor-suppressive programs (5). Senescent cells exhibit distinctive characteristics, such as an enlarged and flattened morphology, senescence-associated β-galactosidase (SA-β-Gal) activity, and altered gene expression (6). Although senescent cells do not divide, they are metabolically active and secrete many proteins to the extracellular space; a vast array of secreted chemokines, cytokines, growth factors, and matrix metalloproteinases (MMPs) comprise the senescence-associated secretory phenotype (SASP) (7). Although senescent cells are beneficial during development, wound repair, and cancer treatment, their accumulation in tissues has been associated with many age-related declines and diseases (8).

Senescent endothelial cells are linked to age-related vascular dysfunction, which leads to arterial stiffness, atherosclerosis, and stroke (9, 10). Proinflammatory or atherosclerotic factors, such as tumor necrosis factor-α, oxidized low-density lipoprotein (ox-LDL), and/or H_2_O_2_, can cause damage in human endothelial cells and promote premature senescence (11, 12). Furthermore, SASP factors secreted from senescent endothelial cells, such as nitric oxide (NO), MMPs, interleukin 1 (IL-1), and IL-8, can trigger several cellular processes, including inflammation, apoptosis, and change in cytoskeletal integrity (13, 14). Accordingly, senescence of endothelial cells induced by different stimuli can influence vascular function and modulate pathogenesis of age-related vascular disease. Therefore, understanding the molecular mechanisms underlying endothelial cell senescence can help establish strategies for preventing senescence-associated vascular diseases.

Fibronectin leucine-rich transmembrane protein 2 (FLRT2) is a member of the highly conserved FLRT family of proteins (15, 16). Structurally, FLRT2 comprises a short intracellular region, a type III fibronectin-like domain, and 10 extracellular leucine-rich domains in humans (17–19). FLRT2 has been reported to promote cell proliferation and mediate cell-cell interactions in chondroprogenitor cells (20) and acts as the predominant ligand of UNC5B during vascular morphogenesis and placental labyrinth formation (21). These reports suggest that FLRT2 is involved in cell cycle regulation and/or vascular development, though relatively little research has focused directly on FLRT2.

Here, we report that FLRT2 prevents endothelial cell senescence and vascular aging through a signaling program that includes integrin subunit beta 4 (ITGB4), mTOR complex 2 (mTORC2), AKT, and p53.

## Results

### FLRT2 levels decline in senescent endothelial cells and in aged vascular tissues.

**Figure 1:**
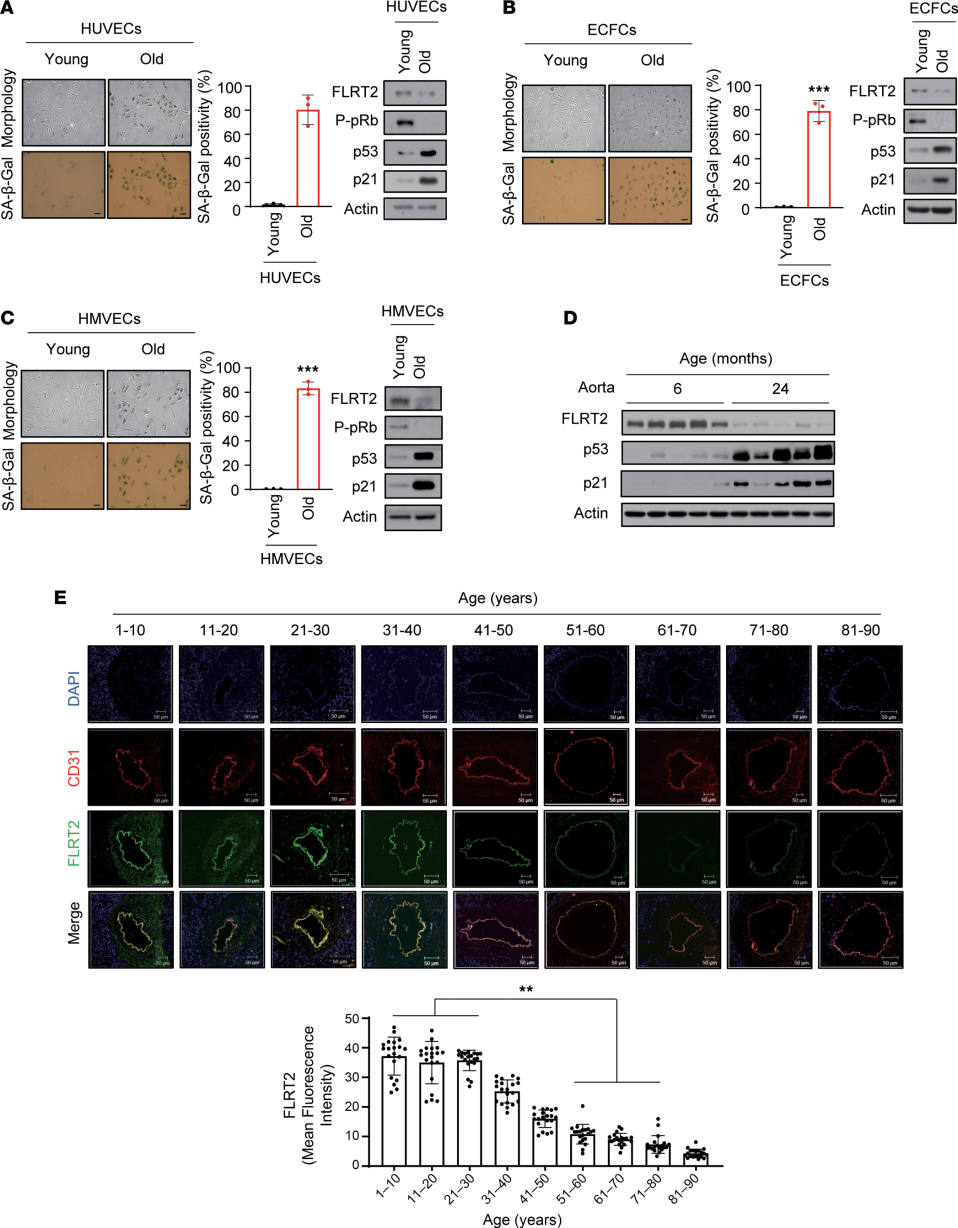
Lower FLRT2 expression levels in senescent endothelial cells and aged rat and human aortic tissues. (**A**–**C**) Young (passage 4) and old (passage 15) human umbilical vein endothelial cells (HUVECs) (**A**), endothelial colony-forming cells (ECFCs) (**B**), and human microvascular endothelial cells (HMVECs) (**C**) were subjected to senescence-associated β-galactosidase (SA-β-Gal) and immunoblot analyses; SA-β-Gal–positive cells were quantified. Scale bar: 10 μm. The values represent mean ± SD (*n* = 3; ***P < 0.001). (**D**) Aortas of 6- and 24-month-old rats were subjected to immunoblot assays. (**E**) Changes in the expression levels of CD31 and FLRT2 in human arterial tissues of each age group (*n* = 20) were detected using immunofluorescence. Scale bar: 50 μm. The values represent mean ± SD (*n* = 20; **P < 0.01). Two-tailed *t* test.

To investigate a possible association between FLRT2 and endothelial cell senescence, we first examined the expression levels of FLRT2 in replicatively senescent human umbilical vein endothelial cells (HUVECs), endothelial colony-forming cells (ECFCs), and human microvascular endothelial cells (HMVECs). Senescence of each endothelial cell type was verified by assessing characteristic morphological changes, higher levels of senescence marker proteins (p53, p21, and unphosphorylated pRb), and increased SA-β-Gal activity (Figure 1, A–C, and Supplemental Figure 1, A–C; supplemental material available online with this article; https://doi.org/10.1172/jci.insight.172678DS1). Importantly, relative to proliferating cells, FLRT2 expression levels declined in all 3 paradigms of senescent endothelial cells. To begin to study whether there might be similar trends in FLRT2 expression in older tissues, where senescent cells accumulate, we examined FLRT2 expression levels in rat and human vascular tissues with increasing age. As shown (Figure 1D and Supplemental Figure 1D), FLRT2 levels decreased dramatically in the aortas of old rats (24 months old) compared with those of young rats (6 months old). In human vascular tissues, p53 and p21 levels increased (Supplemental Figure 2A), while FLRT2 levels decreased in older tissues, especially in people more than 50 years old (Figure 1E and Supplemental Figure 2B). These findings indicate that the expression levels of FLRT2 decline both with senescence in culture and with aging, suggesting connections between the loss of FLRT2 and endothelial cell senescence.

### FLRT2 knockdown induces premature senescence of endothelial cells.

**Figure 2:**
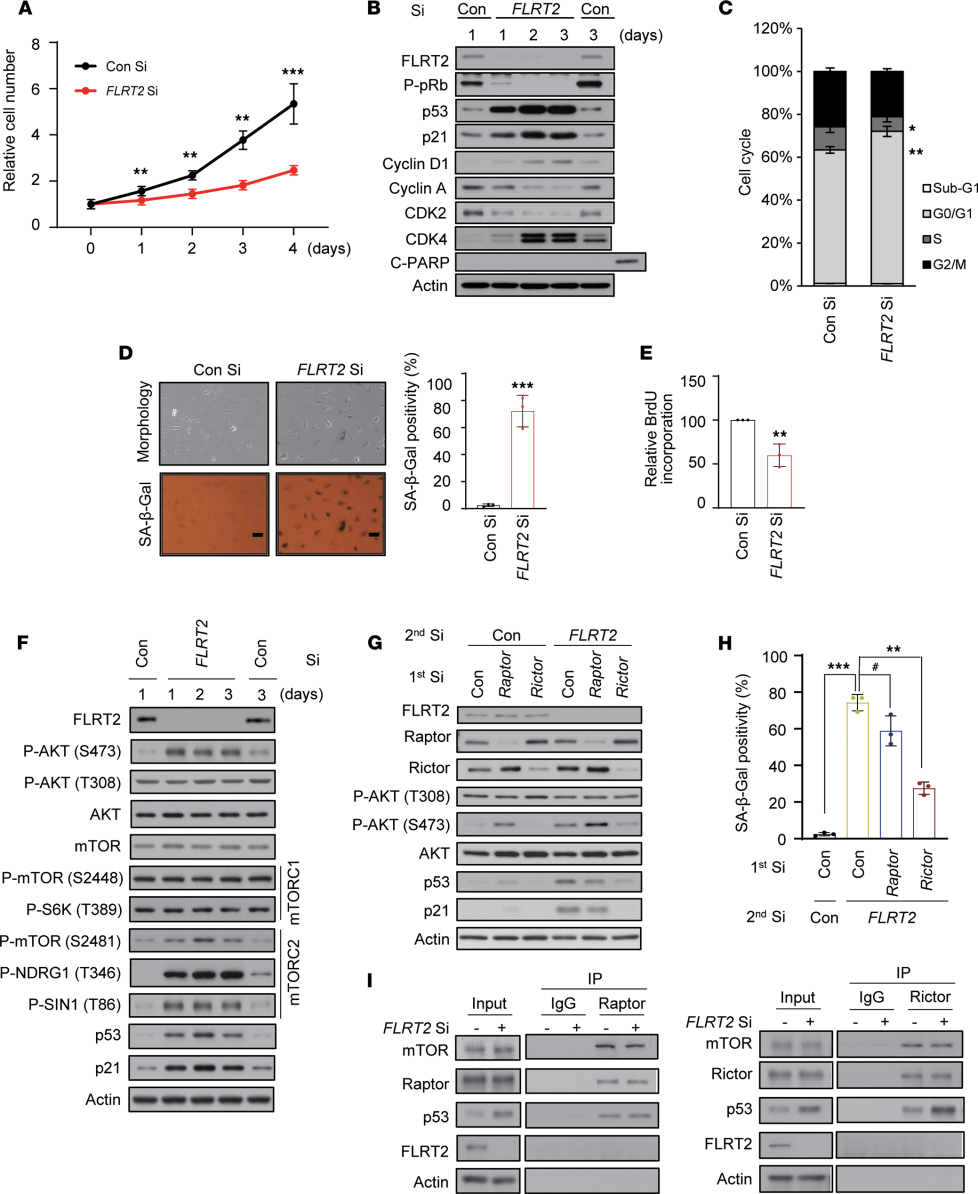
FLRT2 depletion induces cellular senescence through the regulation of mTORC2 in endothelial cells. (**A**–**F**) HUVECs were transfected with 50 nM of control siRNA (Con Si) or FLRT2 siRNA (FLRT2 Si). (**A**) The numbers of viable cells were determined at the indicated days after transfection. (**B**) The cells were harvested at the indicated time points after transfection and subjected to immunoblot analysis. (**C**) Cell cycle distributions were analyzed using flow cytometry at day 3 after transfection. (**D**) Cell morphology and SA-β-Gal activity were assessed at day 3 after transfection, and the percentage of senescent cells was quantified. Scale bar: 10 μm. (**E**) The DNA synthesis rates of siRNA-transfected HUVECs were measured using the 5-bromo-2′-deoxy-uridine (BrdU) incorporation assay. (**F**) Whole-cell lysates were prepared from HUVECs transfected with Con Si and FLRT2 Si at the indicated days after transfection and subjected to immunoblot assays. (**G** and **H**) HUVECs were transfected with Con Si, Raptor Si, or Rictor Si (1st transfection) 6 hours before transfection with Con Si or FLRT2 Si (2nd transfection). At day 2 after transfection, the cells were harvested and subjected to immunoblotting using the indicated antibodies (**G**). SA-β-Gal activity was measured at day 3 after transfection (**H**). (**I**) HUVECs were transfected with Con Si or FLRT2 Si. At day 2 after transfection, cell lysates were subjected to immunoprecipitation with antibodies recognizing Raptor or Rictor, and immunoblot assays were performed with the indicated antibodies. The values represent mean ± SD (*n* = 3; ^#^*P* > 0.05; *P < 0.05; **P < 0.01; ***P < 0.001). Two-tailed *t* test (**A**, **D**, and **E**), 1-way ANOVA (**H**).

To examine the potential role of FLRT2 in endothelial cell senescence, we transfected HUVECs with a small interfering RNA (siRNA) directed at *FLRT2* mRNA (FLRT2 Si). HUVECs in which FLRT2 was silenced proliferated more slowly than control cells (transfected with Con Si) (Figure 2A) and expressed proteins consistent with a phenotype of senescence, including hypophosphorylated pRb and elevated p53 and p21, increased SA-β-Gal positivity, and decreased DNA replication, as evidenced by the relative levels of cells in proliferative cell cycle phases (S and G2/M) and the reduced incorporation of 5-bromo-2′-deoxy-uridine (BrdU) (Figure 2, B–E, and Supplemental Figure 3A). In keeping with earlier reports that senescence decreased the ability of endothelial cells to form tubes in culture (22), the tube-forming ability of FLRT2 was also markedly attenuated in FLRT2-silenced cells (Supplemental Figure 4A). At the same time, FLRT2 knockdown increased the levels of secreted SASP factors (Supplemental Figure 4B). To rule out the possibility of off-target effects by FLRT2 Si, we transfected cells with other FLRT2 siRNAs (FLRT2 Si #2 and #3) and found similar effects on endothelial cell senescence (Supplemental Figure 5). Given the observed rise in p53 and p21 in cells with silenced FLRT2, we studied their involvement in endothelial cell senescence. We discovered that lowering the levels of p53 or p21 abrogated the senescence program elicited by silencing FLRT2 in HUVECs (Supplemental Figure 6). These findings support a model wherein FLRT2 depletion induces cellular senescence in endothelial cells in a p53/p21-dependent fashion.

### FLRT2 regulates endothelial cell senescence through the mTORC2/AKT/p53 pathway.

To further investigate the signaling pathways through which loss of FLRT2 triggers senescence, we treated FLRT2-silenced cells with several kinase inhibitors that have been implicated in senescence: LY294002 (a PI3K inhibitor), rapamycin (an mTORC1 inhibitor), Torin (an mTORC1/2 inhibitor), and PD098059 (an ERK inhibitor). Compared with control cells, treatment with LY29004 or Torin reduced the levels of p53 and p21, as well as SA-β-Gal activity (Supplemental Figure 7, A and B). Thus, we asked whether activation of mTOR and AKT is required for the induction of cell senescence after silencing FLRT2. The effect of FLRT2 silencing on the activities of mTORC1 and mTORC2 in HUVECs is shown in Figure 2F. Silencing FLRT2 triggered the phosphorylations of AKT at serine (S473), mTORC2 complex (S2481), and its substrates, NDRG1 (T346) and SIN1 (T86); by contrast, phosphorylations remained at basal levels for AKT at T308, mTORC1 complex (S2448), and its substrate, S6K (T389) (Figure 2F and Supplemental Figure 8A). Since the functions of mTORC1 and mTORC2 are closely linked to those of Raptor and Rictor, respectively (23, 24), we evaluated the phenotype of cells in which FLRT2 was silenced jointly with Raptor or Rictor. Silencing FLRT2 and Rictor (a subunit of mTORC2) decreased AKT phosphorylation at S473, p53 and p21 accumulation, and SA-β-Gal activity (Figure 2, G and H, and Supplemental Figure 8B). On the other hand, the senescence phenotypes of cells in which FLRT2 and Raptor (a subunit of mTORC1) were silenced were not significantly diminished compared with those of cells in which only FLRT2 was silenced (Figure 2, G and H). We previously found that in phosphatase and tensin homolog (PTEN) loss–induced cellular senescence, mTOR kinase directly binds to p53 and phosphorylates it at serine 15, resulting in p53 activation and p21 accumulation (25). Thus, we examined if mTOR physically associated with p53 cells rendered senescent by silencing FLRT2. Immunoprecipitation (IP) analysis using antibodies against Raptor or Rictor verified an increase in p53 binding to mTORC2 (but not to mTORC1) (Figure 2I). We further conducted an IP assay by using anti-p53 antibody and normalized the result based on the input. Our findings revealed an increased association between Rictor and p53 in FLRT2-silenced cells (Supplemental Figure 9A). This observation was supported by the colocalization of Rictor and p53/phosphorylated p53 (S15) with immunofluorescence staining in FLRT2-silenced cells (Supplemental Figure 9B). Together, these findings suggest that mTORC2 binds to and critically activates p53 levels in endothelial cells rendered senescent by silencing FLRT2.

### Endothelial cell senescence induced by silencing FLRT2 is associated with enhanced ITGB4 activation.

**Figure 3:**
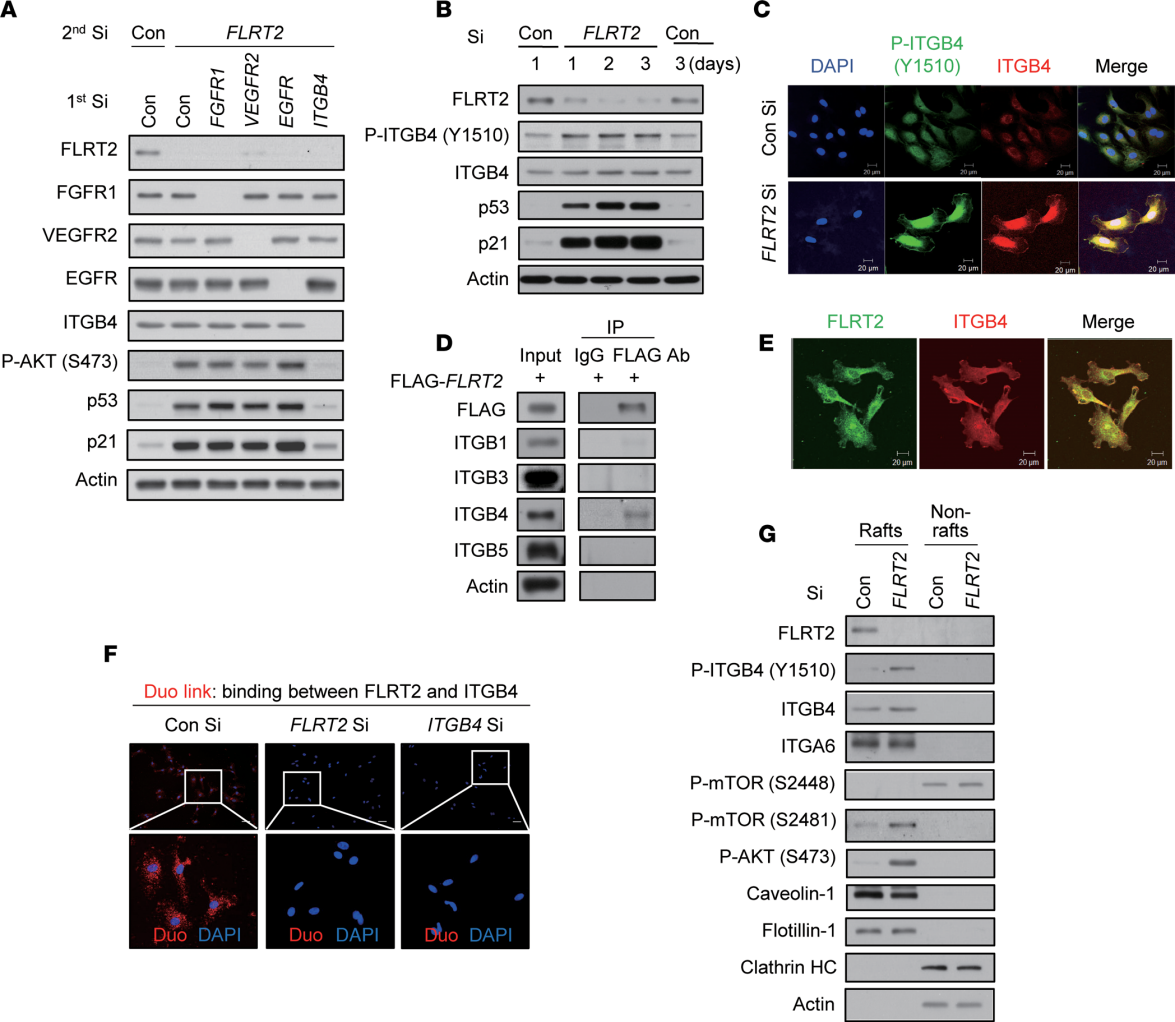
FLRT2 specifically binds ITGB4 and regulates ITGB4 phosphorylation in lipid rafts of the plasma membrane. (**A**) HUVECs were transfected with Con Si, FGFR1 Si, VEGFR2 Si, EGFR Si, or ITGB4 Si (first transfection) 6 hours before transfection with Con Si or FLRT2 Si (second transfection). At day 2 after transfection, the cells were harvested and subjected to immunoblotting using the indicated antibodies. (**B**) Immunoblot assay was performed at the indicated time points after transfection of Con Si or FLRT2 Si. (**C**) Immunocytochemical staining was performed at day 2 after transfection of Con Si or FLRT2 Si. Scale bar: 20 μm. (**D**) HUVECs were transfected with a FLAG-tagged, FLRT2-overexpressing construct. At day 2 after transfection, cell lysates were subjected to immunoprecipitation with anti-FLAG antibody and immunoblotted with the indicated antibodies. (**E**) Immunofluorescence of nonpermeabilized HUVECs that were stained with antibodies recognizing FLRT2 or ITGB4. Scale bar: 20 μm. (**F**) Representative images of individual immunofluorescence staining of FLRT2 and ITGB4 interaction in HUVECs by Duo-link assay. The red dots (FLRT2/ITGB4 interaction) indicate direct interaction. Original magnification, ×40 (top) and ×200 (bottom). Scale bar: 10 μm. (**G**) HUVECs were transfected with Con Si or FLRT2 Si and incubated for 2 days. The lipid rafts were fractionated. Equal volumes of lipid rafts (Rafts; fractions 6–8) and nonlipid rafts (Nonrafts; fractions 2–4) were separated by SDS-PAGE and immunoblotted with the indicated antibodies.

Next, we wondered which molecule plays a role to link senescence signal between FLRT2 and mTORC2/AKT/p53 pathway. To identify a possible candidate protein in this senescence paradigm, we performed a phospho-explorer antibody array analysis in *FLRT2*-silenced cells and control cells: 1,318 well-characterized phosphorylation-specific antibodies were included in this array. After phosphoproteome analysis, changes in protein phosphorylation were calculated by normalization to total protein expression (Supplemental Figure 10A). As shown, among 580 proteins in total, 20.5% of proteins were more than 1.2-fold increased in phosphorylation after silencing *FLRT2* compared with the control silenced group (Supplemental Figure 10A). From proteins with more than 1.2-fold increase in phosphorylation, we found increased phosphorylation of membrane receptors such as fibroblast growth factor receptor 1 (FGFR1), vascular endothelial growth factor receptor 2 (VEGFR2), epidermal growth factor receptor (EGFR), and ITGB4 (Supplemental Figure 10B). The results showed that FGFR1 phosphorylated at tyrosine 766, VEGFR2 phosphorylated at tyrosine 1059, EGFR phosphorylated at tyrosine 1016, and ITGB4 phosphorylated at tyrosine 1510 were upregulated (Supplemental Figure 10B). Previous reports have indicated that proteins in the FLRT family physically interact with FGFRs and modulate the FGFR/ERK signaling pathway (17, 26). Therefore, to investigate whether the FGFR signaling pathway is involved in senescence induced by silencing FLRT2, we examined the roles of FGFR downstream signaling molecules (SRC, ERK, and AKT). We detected a dramatic increase in AKT phosphorylation at S473 but no increase in ERK phosphorylation (Supplemental Figure 10C). When cells were subjected to double siRNA transfections to reduce FLRT2 along with SRC or AKT, the rise in p53 and p21, as well as SA-β-Gal activity, seen after FLRT2 silencing was only abrogated if both FLRT2 and AKT were silenced simultaneously (Supplemental Figure 10D). Subsequently, we investigated the involvement of FGFR1, VEGFR2, EGFR, and ITGB4 in the signaling underlying FLRT2 depletion–induced cellular senescence by reducing them individually in HUVECs using specific siRNAs (Figure 3A and Supplemental Figure 11A). We found that both AKT activation and the rise in p53 and p21 observed in cells rendered senescent by FLRT2 silencing were reversed only by silencing ITGB4. Further experiments verified that ITGB4 was phosphorylated at tyrosine 1510 (Y1510) in the FLRT2-silenced cells (Figure 3, B and C, and Supplemental Figure 11B) and that FLRT2 and ITGB4 physically interacted in FLAG-tagged, FLRT2-overexpressing cells (Figure 3D). Consistent with these observations, we found colocalization of FLRT2 and ITGB4 by immunofluorescence staining of HUVECs (Figure 3E) and verified the interaction between FLRT2 and ITGB4 by using the Duo-link assay (Methods and Figure 3F). Moreover, increased ITGB4 phosphorylation at Y1510 was observed in lipid rafts of the plasma membrane of FLRT2-silenced cells (Figure 3G and Supplemental Figure 11C). These results suggest that FLRT2 associates with and prevents the activation of ITGB4 in lipid rafts and further suggest that FLRT2 depletion may promote cellular senescence by facilitating the activation of ITGB4.

**Figure 4:**
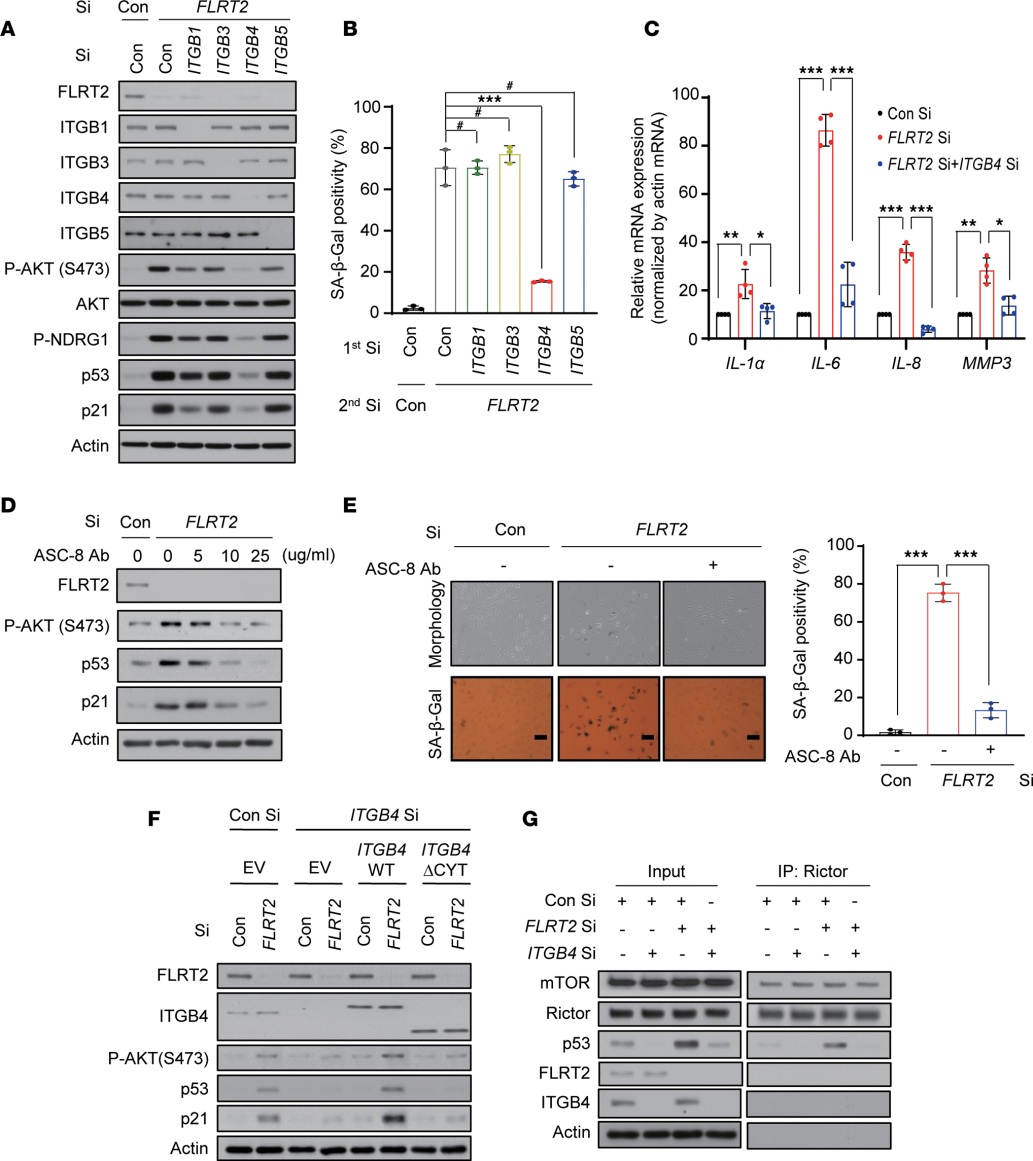
Signaling through ITGB4, mTORC2, and AKT is required for a senescence program triggered by FLRT2 depletion. (**A** and **B**) HUVECs were transfected with Con Si, ITGB1 Si, ITGB3 Si, ITGB4 Si, or ITGB5 Si (first transfection) 6 hours before transfection with Con Si or FLRT2 Si (second transfection). At day 2 after transfection, cells were harvested and subjected to immunoblotting (**A**). At day 3 after transfection, SA-β-Gal activity was examined (**B**). (**C**) HUVECs were transfected with Con Si or FLRT2 Si, and reverse transcription (RT) followed by real-time quantitative polymerase chain reaction (qPCR) (RT-qPCR) analysis was carried out 2 days after transfection. (**D** and **E**) HUVECs were transfected with Con Si or FLRT2 Si, followed by treatment with an inhibitor of ITGB4 (ASC-8). Immunoblot assay (**D**) and SA-β-Gal assay (**E**) were performed at days 2 and 3 after transfection, respectively. Scale bar: 10 μm. (**F**) HUVECs were transfected with Con Si or ITGB4 Si 1 day before transfection with empty vector (EV), a vector encoding wild-type ITGB4 (WT), or a vector encoding a truncated mutant of ITGB4 lacking the residues downstream of amino acid 1355 (ΔCYT), followed by transfection with Con Si or FLRT2 Si. At day 2 after transfection, the cells were subjected to immunoblotting analysis. (**G**) HUVECs were transfected with Con Si or ITGB4 Si. At 6 hours after transfection, one-half of each group was transfected with Con Si, while the other half was transfected with FLRT2 Si. At day 2 after transfection, cell lysates were subjected to immunoprecipitation with anti-Rictor antibody. Mean ± SD (*n* = 3; ^#^*P* > 0.05; *P < 0.05; **P < 0.01; ***P < 0.001). One-way ANOVA.

To investigate the possibility of other β-integrin involvements in senescence induced by silencing FLRT2, we individually silenced ITGB1, -3, and -5, which are known to be expressed in endothelial cells (27). As shown, only silencing ITGB4 significantly suppressed the increase in levels and/or activity of mTORC2, AKT, p53, p21, and SA-β-Gal (Figure 4, A and B, and Supplemental Figure 12A) triggered by FLRT2 depletion, as well as the increased abundance of mRNAs encoding the SASP factors IL-1A (IL-1α), IL-6, IL-8, and MMP3 (Figure 4C) in cells rendered senescent by silencing FLRT2. Treatment with increasing doses of ASC-8, an ITGB4 inhibitor, progressively decreased the phosphorylation of AKT and the accumulation of p53 and p21 in FLRT2-silenced HUVECs (Figure 4D and Supplemental Figure 12B) and markedly reduced cell senescence elicited by silencing FLRT2 (Figure 4E). This suggests that ITGB4 plays a specific role in the FLRT2-mediated pathway that leads to endothelial cell senescence.

Next, cells in which ITGB4 and FLRT2 were silenced were transfected with vectors that expressed wild-type (WT) ITGB4 or mutant ITGB4 ΔCYT (which lacked the tyrosine phosphorylation site present in the cytoplasmic tail; ref. 28) as shown in Figure 4F. In these cells, ITGB4 WT induced AKT phosphorylation and expression of p53 and p21, but ITGB4 ΔCYT did not (Figure 4F and Supplemental Figure 12C). We also transfected ITGB4 WT and ITGB4 ΔCYT into human aortic endothelial cells (HAECs), which do not express endogenous ITGB4 (29). In these cells, transfection with ITGB4 WT markedly increased AKT, p53, and p21 and other senescence phenotypes in HAECs, while ITGB4 ΔCYT expression did not (Supplemental Figure 13). To further verify whether ITGB4 might require the physical interaction between mTORC2 and p53 in FLRT2 depletion–induced cellular senescence, we immunoprecipitated Rictor in the presence or absence of ITGB4 in FLRT2-silenced cells (Figure 4G). We found that Rictor physically associated with p53 in FLRT2-silenced cells in the presence of ITGB4, while the binding of Rictor to p53 was completely inhibited in cells in which FLRT2 and ITGB4 were silenced, indicating that the binding of p53 to mTORC2 is mediated by ITGB4. These findings provide evidence that ITGB4 is a key player in the program of endothelial cell senescence triggered by FLRT2 silencing and that mTORC2, AKT, and p53 signaling pathways are downstream effectors in this paradigm.

### Knockdown of FLRT2 induces senescence in mouse aorta and vein endothelial cells.

**Figure 5:**
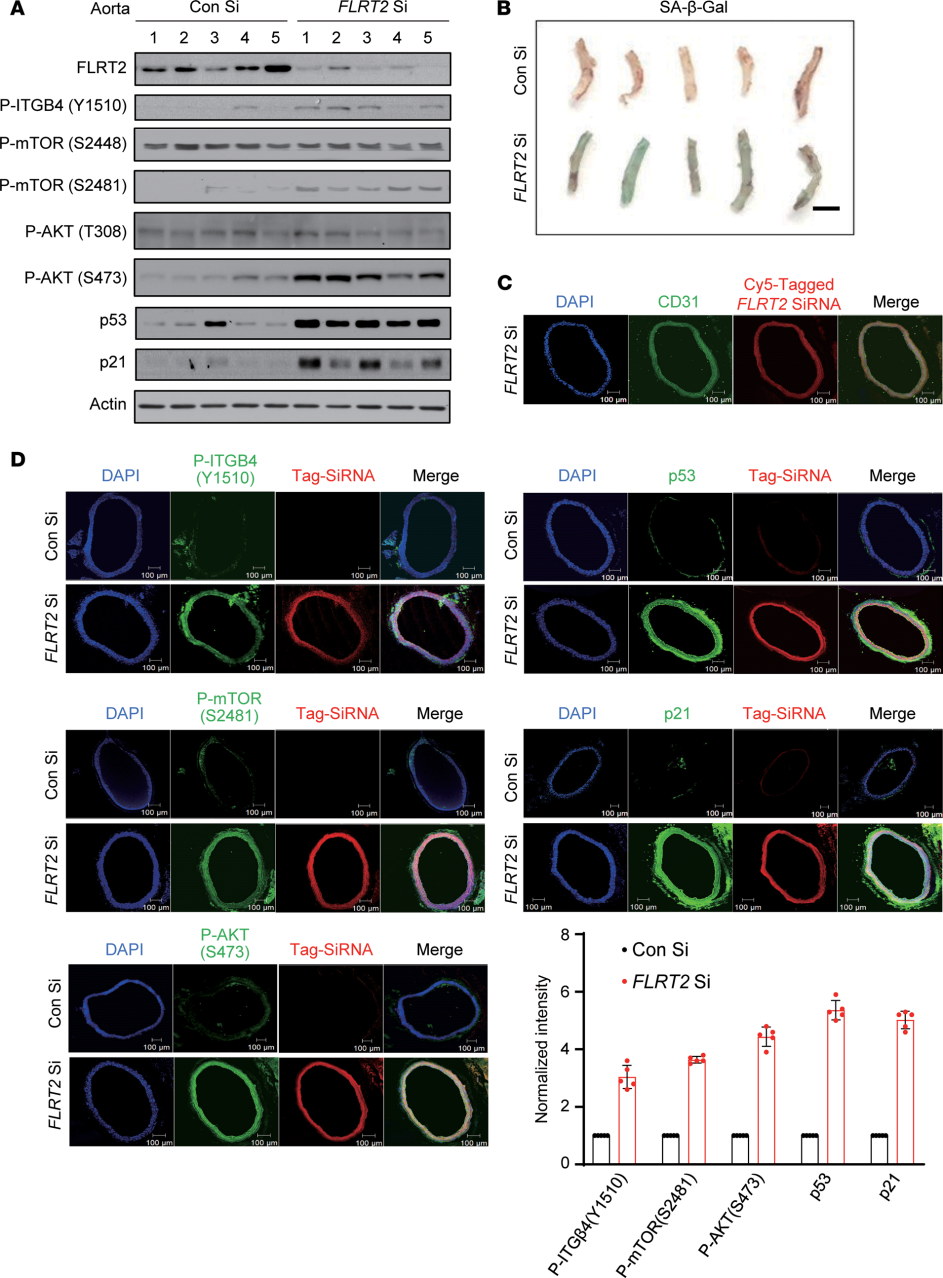
FLRT2 silencing promotes aortic endothelial cell senescence. (**A**) Representative immunoblot images obtained from aortic lysates of mice injected intravenously with Con Si or FLRT2 Si (*n* = 5). (**B**) Images of mouse aortas assayed for SA-β-Gal activity. Scale bar: 2 mm. (**C**) Fluorescence micrographs of aortic cross sections obtained from mice injected intravenously with Cy5-tagged FLRT2 Si. Nuclei are stained with DAPI (blue). Red and green signals show Cy5-tagged FLRT2 Si and the endothelial marker CD31, respectively. Merged image indicates siRNA uptake by the endothelium. Scale bar: 100 μm. (**D**) Immunostaining for p-ITGB4 (Y1510), p-mTOR (S2481), p-AKT (S473), p53, and p21 in aortas obtained from mice injected with siRNAs. Scale bar: 100 μm. Quantitative data are shown in the graph.

To begin to explore the biological significance of our findings in vivo, we examined the effects of FLRT2 knockdown on senescence phenotypes in mouse vascular tissues. Using a transient silencing approach (30), we reduced aortic FLRT2 expression by injecting Cy5-tagged FLRT2 Si (or Con Si in control groups), along with an in vivo transfection reagent (Methods), into the tail veins of mice (Figure 5A). After lowering FLRT2 levels, aorta tissues revealed increased phosphorylation of ITGB4 at Y1510, mTOR at S2481, and AKT at S473 and higher abundance of p53 and p21 (Figure 5A and Supplemental Figure 14A), along with higher levels of SA-β-Gal signal, as compared with the Con Si group (Figure 5B). Delivery of Cy5-tagged FLRT2 Si to the vasculature and uptake by the endothelium were verified by CD31 immunostaining (Figure 5C). To precisely determine the localization of FLRT2 in mouse blood vessels, we investigated its colocalization with the endothelial cell marker CD31, tunica media marker α–smooth muscle actin (α-SMA), and pericyte marker neuron glial antigen-2 (NG2) in aortic cross sections. Our results demonstrated that the FLRT2 signal colocalized with the CD31 signal but exhibited no overlap with α-SMA or NG2 (Supplemental Figure 15A). To validate this finding, we performed Western blot analysis in 2 mouse cell lines, C166 (mouse endothelial cells) and mouse aortic vascular smooth muscle cells (MOVAS), and found that FLRT2 was exclusively expressed in C166 mouse endothelial cells (Supplemental Figure 15B). Finally, through fluorescence microscopy, we verified that FLRT2 silencing induced phosphorylations of ITGB4, mTORC2, and AKT, along with increased expression levels of p53 and p21 in arterial endothelial cells (Figure 5D).

**Figure 6:**
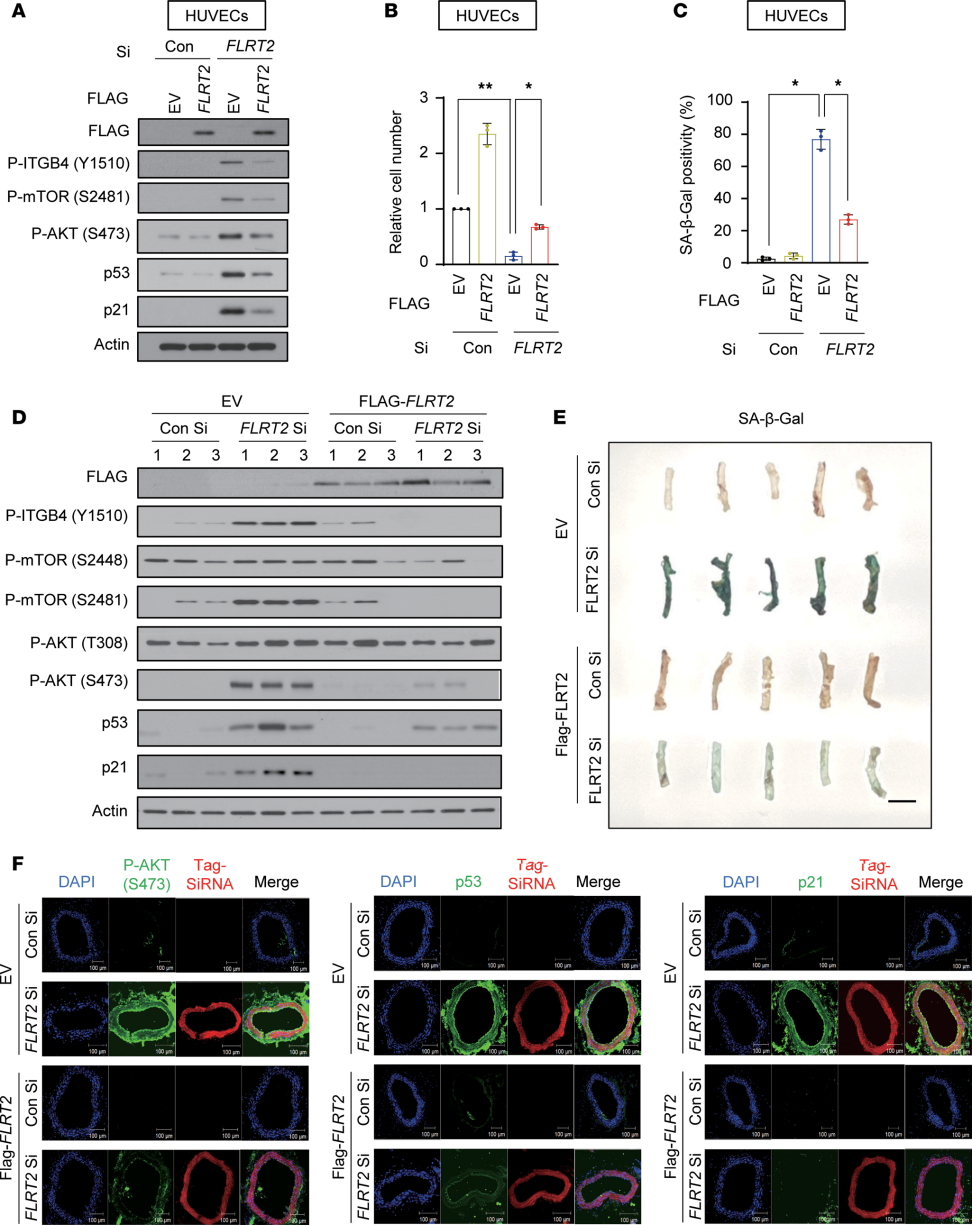
Overexpression of FLRT2 rescues the vascular aging effect of silencing FLRT2 on endothelial cell senescence. (**A**–**C**) HUVECs exogenously expressing empty vector or Flag-FLRT2 were transfected with Con Si or FLRT2 Si. Immunoblot assay was performed with the indicated antibodies (**A**), and relative cell number (**B**) and SA-β-Gal activity (**C**) were assessed at 3 days after siRNA transfection. The values represent the mean ± SD (*n* = 3; *P < 0.05; **P < 0.01). One-way ANOVA. (**D**) Representative immunoblot images obtained from aortic lysates of mice injected intravenously with Con Si, FLRT2 Si, empty vector, or Flag-FLRT2 plasmid (*n* = 5). (**E**) Image of SA-β-Gal–stained aortas of mice. Scale bar: 5 mm. (**F**) Immunostaining for p-AKT (S473), p53, and p21 in aortas of mice. Scale bar: 100 μm.

In addition, in the veins of mice injected with FLRT2 Si, we observed increased SA-β-Gal activity and elevated signaling through ITGB4, mTORC2, AKT, p53, and p21 (Supplemental Figure 16, A–D). We next examined whether FLRT2 overexpression in FLRT2-silenced cells diminished the senescence phenotype promoted by FLRT2 silencing. The endothelial cell senescence traits induced by FLRT2 silencing (which elevated phosphorylation of ITGB4 at Y1510, mTOR at S2481, and AKT at S473; increased p53 and p21 levels and SA-β-Gal activity; and decreased cell proliferation) were evidently diminished by FLRT2 overexpression in cultured endothelial cells (Figure 6, A–C, and Supplemental Figure 17A). To test whether overexpression of FLRT2 rescues the FLRT2 Si–induced senescence in vivo, we injected Cy5-tagged FLRT2 Si, which is specific to the *FLRT2* 3′UTR (to avoid silencing the ectopic *FLRT2* mRNA), or Con Si with either FLRT2 expression vector or empty vector into the tail veins of mice, along with an in vivo transfection reagent. Consequently, when we overexpressed FLRT2 in FLRT2-silenced mice, which were generated using in vivo-jetPEI (Polyplus transfection), we found that the senescence phenotypes, including increased SA-β-Gal activity, phosphorylation of AKT at S473, and expression levels of p53/p21 showing in FLRT2-silenced mice, were rescued in the aortas of mice (Figure 6, D and E, and Supplemental Figure 17B). Immunostaining of phosphorylated AKT (S473), p53, and p21 was also evidently decreased to the control level in aorta tissues of mice (Figure 6F). Collectively, our findings lend strong support to a model whereby FLRT2 regulates endothelial cell senescence through a signaling paradigm involving ITGB4, mTORC2, AKT, p53, and p21 in cultured endothelial cells and mouse vascular tissue.

## Discussion

There is limited information about the functional roles of FLRT2 in cultured cells and in mice, although there is evidence that it is implicated in cardiovascular function. Ablation of FLRT2 in mouse embryos disrupted heart formation and led to cardiac insufficiency (31) whereas another study reported that FLRT2 is expressed in endothelial cells in the placental labyrinth and that mice lacking FLRT2 in endothelial cells exhibited embryonic lethality with systemic congestion and hypoxia (21). Here, we first showed that FLRT2 expression declined in senescent endothelial cells and in aged rat and human arterial tissues (Figure 1). We then observed that depleting FLRT2 in endothelial cells induced cellular senescence (Figure 2). During mouse embryogenesis, FLRT proteins reportedly regulate FGF signaling in many tissues by interacting with a signaling receptor (25). We previously reported that signaling through FGFR and AKT promotes the expression of p53 and p21 during senescence in cancer cells (32). Here, however, we observed that FGFR1 and downstream signaling molecules SRC and ERK were not involved in FLRT2 depletion–induced endothelial cell senescence (Supplemental Figure 6).

ITGB4, a cell surface laminin receptor (33, 34), is distinguished by its remarkably large cytoplasmic domain, which primarily contributes to mediating signaling pathways (35). Previous studies have indicated that ITGB4 is implicated in endothelial cell senescence. Specifically, it mediates endothelial cell senescence by suppressing Ca^2+^-independent PC-PLC and increasing the levels of p53 and reactive oxygen species (36). Additionally, 3BDO has been shown to inhibit endothelial cell senescence, downregulating the expression of ITGB4 (37).

In recent decades, there has been a growing interest in the role of mTOR as a regulator of life span and aging (38–40). Most studies have focused on the impact of the mTORC1/ribosome protein S6 kinase beta-1 (S6K1) signaling pathway on senescence. Inhibition of mTORC1 or deletion of the mTORC1 substrate S6K1 has been associated with increased life span (38–40). In the context of endothelial senescence, mTORC1/S6K1 signaling has been found to causatively contribute to endothelial nitric oxide synthase uncoupling (41), as well as the expression of coagulation factor tissue factor (42, 43). Although the role of mTORC1 in endothelial senescence is well documented, the regulatory mechanisms and functions of mTORC2 are less understood. Limited published reports suggest that activation of mTORC2/AKT by arginase 2 accelerates endothelial cell senescence and atherosclerotic plaque formation by activating mTORC1/S6K1, thereby suppressing endothelial autophagy (44). Prolonged activation of mTORC2/AKT signaling due to diet-induced obesity has also been linked to vascular senescence (45). Yang et al. observed an increase in mTORC2 activity during both replicative senescence and H_2_O_2_-induced premature senescence in HUVECs, with concomitant reduction of nuclear factor erythroid-2-related factor 2 (NRF2) mRNA expression (46). Inhibition of mTORC2 and AKT signaling through siRNA knockdown of Rictor and treatment with an AKT inhibitor, respectively, resulted in the attenuation of HUVECs’ senescence. Rictor knockdown also reversed the suppression of NRF2 mRNA expression. Yang et al. concluded that mTORC2/AKT mediates HUVECs’ senescence via the suppression of NRF2 (46). We recently reported that the activation of ITGB4 is essential for radiation-induced cellular senescence in A549 lung cancer cells (47) and that mTOR kinase is a key activator of p53 in senescence triggered by loss of PTEN in cancer cells (26). However, the underlying mechanism through which ITGB4 and mTORC2 contribute to endothelial cell senescence is not well understood.

In this study, we identified ITGB4 as an important intermediary molecule for the activation of mTORC2, but not mTORC1, in endothelial senescence and vascular aging (Figures 2–4). Our data suggest that among ITGB family members, ITGB4 plays a specific role in mediating endothelial cell senescence by linking signals from the transmembrane protein, FLRT2, to the mTORC2/AKT/p53 signaling paradigm. A physical association between FLRT2 and ITGB4 in lipid rafts of the plasma membrane critically prevented ITGB4 phosphorylation and thereby maintained ITGB4 in an inactive state (Figure 3). If FLRT2 did not properly inhibit ITGB4 activation, persistent activation of signaling through mTORC2/AKT/p53 induced endothelial cell senescence, as shown in aortic and venous tissues of the FLRT2-knockdown mouse model (Figure 5 and Supplemental Figure 16), and by the rescue of the effects of FLRT2 loss on endothelial cell senescence and vascular aging when FLRT2 was overexpressed (Figure 6). Furthermore, we have demonstrated that FLRT2 plays a crucial role in protecting against vascular aging in human arterial tissues across various age groups, ranging from the tenth decade to the eighties (Figure 1E). Our findings indicate that an interaction between FLRT2 and ITGB4 in lipid rafts prevents endothelial cell senescence and vascular aging by acting through mTORC2, AKT, p53, and p21.

Vascular endothelial cells can undergo premature senescence upon exposure to agents that are risk factors for vascular diseases. Exposure of endothelial cells to low or high glucose promotes the expression of senescence markers, such as SA-β-Gal, p16, and inducible NOS, and decreases the expression of endothelial NOS (48, 49). Other stimuli, such as ox-LDL, homocysteine, ceramide, and angiotensin II (Ang II), promote endothelial cell senescence through various mechanisms (50–54). For example, Ang II induces endothelial cell senescence by activating the mitogen-activated protein kinases (54), and ox-LDL promotes HUVECs’ senescence by lowering sirtuin1 production (55). The induction of senescence increases the susceptibility of endothelial cells to pathological stresses, which results in endothelial injury and consequently promotes age-related vascular diseases, such as cardiovascular diseases (coronary artery disease, atherosclerosis, and hypertension), peripheral vascular diseases, diabetic retinopathy, renal vascular diseases, and microvascular diseases (50, 56). Therefore, we propose that therapeutic regimens capable of regulating upstream molecules FLRT2 and ITGB4 in this endothelial cell senescence paradigm could be developed as a novel strategy for the prevention of vascular aging and age-related vascular diseases.

## Methods

### Sex as a biological variable.

Our study examined male mice because male animals exhibited less variability in phenotype.

### Cell culture.

HUVECs, ECFCs, HMVECs, and HAECs were cultured in endothelial cell growth basal medium-2 (Lonza) supplemented with 2% fetal bovine serum, human FGF-β, VEGF, R3-insulin growth factor-1, human EGF, hydrocortisone, ascorbic acid, heparin, gentamicin, and amphotericin-B (Lonza). HMVECs were cultured in microvascular endothelial cell growth medium BulletKit (Lonza). C166 cells and MOVAS were cultured in DMEM (WelGENE) supplemented with 10% FBS (Lonza) and 1% penicillin and streptomycin (WelGENE). HUVECs, HMVECs, and HAECs were purchased from Lonza. ECFCs were gifted by Man Ryul Lee (Soonchunhyang University, Cheonan, Korea).

### Reagents and antibodies.

Antibodies recognizing phospho-pRb (S807/811, catalog 9308), cleaved PARP (catalog 9541), phospho-SRC (Y416, catalog 2101), p21 (catalog 2947, catalog 64016), mTOR (catalog 2972), phospho-mTOR (S2448, catalog 2971S), phospho-mTOR (S2481, catalog 2974S), phospho-ERK1/2 (catalog 9101), phospho-AKT (T308, catalog 13038), phospho-AKT (S473, catalog 4060), phospho-NDRG1 (T346, catalog 5482), phospho-p70S6K (T389, catalog 9206), phospho-SIN1 (T86, catalog 14716S), and phospho-p53 (S15, catalog 9284) were from Cell Signaling Technology. Antibodies recognizing flotillin-1 (catalog sc-74566), cyclin D1 (catalog sc-8396), cyclin A (catalog sc-271682), CDK2 (catalog sc-6248), CDK4 (catalog sc-23896), AKT (catalog sc-5298), integrin β1 (catalog sc-374429), integrin β3 (catalog sc-365679), and integrin β5 (catalog sc-398214) were from Santa Cruz Biotechnology. Antibodies recognizing clathrin (catalog 610500) and caveolin-1 (catalog 610493) were purchased from BD Biosciences. Antibodies recognizing p53 (catalog NCL-L-p53-DO7), actin (catalog ab8226), and CD31(catalog MA3105) were from Leica Biosystems, Abcam, and Thermo Fisher Scientific, respectively. Antibodies recognizing integrin β4 (catalog ab182120), integrin α6 (catalog ab181551), Raptor (catalog ab2280), Rictor (catalog ab2140), and FLRT2 (catalog ab154023) were from Abcam, while an antibody recognizing phospho-integrin β4 (Y1510, catalog CBP1427) was from Assay Biotechnology. The anti–integrin β4 antibody (clone ASC-8, catalog MAB2059) for blocking integrin β4 activity was purchased from MilliporeSigma. Rapamycin, LY294002, and PD098059 were purchased from MilliporeSigma. Torin was purchased from Selleckchem.

### SA-β-Gal staining.

Cells were washed with 1× phosphate-buffered saline (PBS), fixed in 3.7% formaldehyde for 5 minutes, and washed with PBS. The fixed cells were then incubated with a solution containing 1 mg/mL of 5-bromo-4-chloro-3-indolyl B-d-galactoside, 40 mM citric acid/sodium phosphate (pH 6.0), 5 mM potassium ferrocyanide, 5 mM potassium ferricyanide, 150 mM NaCl, and 2 mM MgCl_2_ for 16 hours at 37°C. The cells were then washed and imaged using a microscope (Olympus CKX41).

### Human arterial tissue samples.

Human vascular tissue sections of the splenic arteries were obtained from Yeungnam University Hospital (Korea) (aged 1–90 years, grouped in 10-year age intervals; *n* = 20 for each group).

### Immunoblotting analysis.

The cells were lysed using radioimmunoprecipitation assay lysis buffer containing protease inhibitors (Roche) and phosphatase inhibitors (MilliporeSigma). Equal amounts of proteins were subjected to SDS-PAGE. The resolved proteins were transferred to a nitrocellulose membrane, which was then blocked with 3% nonfat dried milk or 3% bovine serum albumin (BSA) and incubated with the primary antibodies at 4°C overnight. Next, the membrane was incubated with horseradish peroxidase–conjugated secondary antibodies (Cell Signaling Technology catalog 7076 and catalog 7074) for 1 hour. The protein bands were developed using an enhanced chemiluminescence reagent (Thermo Fisher Scientific) and visualized after exposure to x-ray film (Agfa Gevaert NV).

### IP.

The cell lysates were prepared using a 1% NP-40 lysis buffer (25 mM Tris-HCl at pH 7.5, 150 mM NaCl, and 1% NP-40) containing protease inhibitors (Roche) and phosphatase inhibitors (MilliporeSigma). The lysates were precleared with Sepharose beads coupled with Protein G or A (MilliporeSigma), and the indicated antibodies were added and tumbled with Sepharose beads coupled with Protein G or A. The immune complexes were then subjected to SDS-PAGE, followed by immunoblotting.

### Cell viability.

Cells were seeded in 60 mm dishes, cultured for 24 hours, and treated with conditioned media (CM) as indicated in the figures for 3 days. Next, the cells were trypsinized and harvested. Cell viability was measured using the trypan blue exclusion assay; cell suspensions were diluted (1:1) with 0.4% trypan blue (Gibco) and counted using a hemocytometer under a microscope (Olympus CKX41).

### Cell cycle analysis.

Cells were harvested by trypsinization, fixed in 95% ethanol, washed with PBS, resuspended in 1 mL PBS containing 1 mg/mL RNase and 50 mg/mL propidium iodide (MilliporeSigma), and incubated in the dark for 30 minutes. The distribution across cell cycle compartments was analyzed using a FACSCalibur flow cytometer (BD Biosciences).

### BrdU incorporation assay.

Cell proliferation was measured using a BrdU cell proliferation assay kit (Roche Applied Science) following the manufacturer’s instructions. BrdU incorporation was determined by measuring the absorbance at 450 nm using a spectrophotometer (Biotek Power Wave XS).

### RNA interference.

Cells were transfected with siRNA duplexes using RNAiMAX (Invitrogen). The sequences of the siRNAs (Bioneer Inc.) were as follows: Con Si: 5′ CCUACGCCACCAAUUUCGUdTdT 3′, FLRT2 Si: 5′ GAGCUUGGUAAAUGUCACUdTdT 3′, FLRT2 Si #2: 5′ CACUCUACACUAUACAGUAdTdT 3′, FLRT2 Si #3: 5′ GCAUUUCUAAAGGCCAUUUdTdT 3′, Mouse FLRT2 Si: 5′ [Cyanine5]CACUCUACACUAUACAGUAdTdT 3′, p53 Si: 5′ CACUACAACUACAUGUGUAdTdT 3′, p21 Si: 5′ CUGUACUGUUCUGUGUCCUdTdT 3′, SRC Si: 5′ GGCUGAGGAGUGGUAUUUUdTdT 3′, AKT Si: 5′ GACAACCGCCAUCCAGACUdTdT 3′, Raptor Si: 5′ CACCTCACTTTATTTCCATdTdT 3′, Rictor Si: 5′ ACUUGUGAAGAAUCGUAUCdTdT 3′, ITGB1 Si: 5′ CAGACAUCAUUCCAAUUGUdTdT 3′, ITGB3 Si: 5′ CAGAUGUCAUUCCAUAUCAdTdT 3′, ITGB4 Si: 5′ GACUUCGUGUGCGGACAGUdTdT 3′, and ITGB5 Si: 5′ CUGUUGAAGGUACAUCGUUdTdT 3′.

### Plasmid transfection.

Plasmids were transfected using Lipofectamine 2000 reagent (Invitrogen), following the manufacturer’s instructions. The plasmids expressing pRK5 beta4 (plasmid 16037) and pRK5 beta4 1355 T (ΔCYT) (plasmid 16038) plasmid were purchased from Addgene. The plasmids expressing FLRT2 (NM_013231) Human Tagged ORF Clone and FLRT2 (NM_201518) Mouse Tagged ORF Clone were purchased from OriGene.

### RT-qPCR.

Total RNA was extracted using the TRIzol reagent (Invitrogen), following the manufacturer’s instructions, and resuspended in diethyl pyrocarbonate–treated water. The RNA was subjected to RT using a cDNA synthesis kit (Bio-Medical Science Co. Ltd). The cDNA was then subjected to real-time qPCR analysis using predesigned gene-specific primers purchased from Bioneer Co., Ltd. and iQ SYBR Green Supermix (Bio-Rad Laboratories) in a CFX Connect Real-Time PCR Detection System (Bio-Rad Laboratories).

### Phosphoprotein profiling.

The Phospho Explorer Antibody Microarray, which was produced by Full Moon Biosystems Inc., was used to examine changes of protein phosphorylation. After conducting the antibody array experiment, the slide was scanned on an Axon GenePix array scanner. Then we analyzed the images using GenePix Pro 6.0. The fluorescence signal for each antibody was gathered from the fluorescence intensity of its respective antibody spot. The phosphorylation ratio was calculated as follows: phosphorylation ratio = phosphorylated value/unphosphorylated value.

### Tube formation assay.

Matrigel (BD Biosciences) was polymerized (200 μL/well of a 48-well tissue culture plate) for 30 minutes at 37°C. Trypsinized HUVECs (4 × 10^4^) were resuspended in 200 μL of CM and seeded into each well. The cells were cultured for 16 hours, and images of the tubes were captured using a microscope. The number of branch points and branches was counted, and the tube area was measured using the Fuji Multi Gauge V2.3 software.

### Immunofluorescence staining.

The cells were fixed with 3.7% formaldehyde for 15 minutes, permeabilized with 0.1% Triton X-100 for 15 minutes, and blocked using 3% BSA in PBS for 1 hour. Next, the cells were incubated with the primary and fluorescein-conjugated secondary antibodies for 1 hour at room temperature. Immunofluorescence was examined using an LSM 510 META laser scanning microscope (ZEISS).

### The Duo-link proximity ligation assay.

To assess possible physical interaction between FLRT2 and ITGB4, HUVECs were subjected to Duo-link assay with anti-FLRT2 (catalog ab154023, Abcam) and anti-ITGB4 (catalog ab182120, Abcam) as primary antibodies, following the manufacturer’s instructions (MilliporeSigma). Fluorescence images of the cells were obtained under an LSM 510 META laser scanning microscope.

### Isolation and characterization of lipid rafts.

HUVECs were lysed with 1 mL of lysis buffer (1% Triton X-100, 25 mM HEPES at pH 6.5, 150 mM NaCl, 1 mM EDTA, 1 mM phenylmethylsulfonyl fluoride, and protease inhibitor cocktail). Equal amounts of proteins were subjected to discontinuous sucrose gradient ultracentrifugation (40%, 30%, and 5%) using an SW41 Ti rotor (260,809*g*) for 16 hours at 4°C. The sucrose gradients were fractionated into 13 fractions, including the pellet. An opaque buoyant band corresponding to the lipid rafts was collected from the interface between the 30% and 5% sucrose gradients. Aliquots of fractions collected from the bottom of the gradient were analyzed using immunoblotting. The protein concentration was determined using the bicinchoninic acid method (Thermo Fisher Scientific), following the manufacturer’s instructions.

### Immunohistochemistry.

The tissues were cryosectioned into 20 μm–thick sections, fixed with 3.7% formaldehyde, and permeabilized with 0.01% Triton X-100 for 15 minutes. The samples were then blocked with 3% BSA for 1 hour at room temperature and incubated with rat anti-CD31 (catalog MA3105, Thermo Fisher Scientific) and anti-FLRT2 (catalog ab154023, Abcam) diluted (1:50) in blocking solution overnight at 4°C. The sections were washed with PBS and incubated with Alexa Fluor 594 goat anti-hamster IgG (catalog 127-025-160, Jackson ImmunoResearch Laboratories) and Alexa Fluor 488 goat anti-rabbit (catalog 127-095-160, Jackson ImmunoResearch Laboratories) diluted (1:50) in blocking solution for 1 hour at room temperature. The sections were washed thrice with PBS and mounted on microscopy slides.

### Animal experiments.

A total of 50 μg of Cy5–tagged mouse FLRT2 Si or Flag-tagged FLRT2 plasmid was mixed with in vivo-jetPEI (Polyplus transfection), following the manufacturer’s instructions. Cy5–tagged mouse FLRT2 Si was used to assess siRNA distribution in mouse aorta (thoracic) or vein (vena cava). The solution was vortexed gently and left for 15 minutes at room temperature. The mixture was injected into the tail veins of 8-week-old male mice (Orient Bio Inc.) 4 times over 14 days, and the treated mice were sacrificed for immunoblotting, SA-β-Gal staining, and immunofluorescence. For investigating the expression levels of FLRT2 in rat vascular tissues with increasing age, carotid arteries of 6- and 24-month-old rats were a gift from Hae Young Chung (Pusan National University, Pusan, Korea).

### Statistics.

All data are expressed as mean ± SD. The data were analyzed using 1-way ANOVA or 2-tailed *t* test. The differences were considered significant at *P* < 0.05.

### Study approval.

All animal care and experimental procedures were approved by the Inha University Institutional Animal Care and Use Committee (Approval Number INHA-200917-722-1). Human experimental protocols were approved by Internal Review Board of Yeungnam University Hospital from 1995 to 2012 (YUMC 2015-01-017).

### Data availability.

All individual data values represented in graphs are available in the Supporting Data Values file.

## Author contributions

HJH investigated; HJH and JSL wrote the original draft; HJH, JSL, DK, JRK, YGK, HJP, ABH, and MG reviewed and edited the manuscript; HJH performed validation; HJH performed visualization; DK, JRK, JKR, YGK, and HJP performed conceptualization; JHC and JRK provided resources; JSL performed data curation; MG and JSL supervised; JSL performed project administration; JSL performed funding acquisition.

## Supplementary Material

Supplemental data

Unedited blot and gel images

Supporting data values

## Version 1 04/08/2024

Electronic publication
