# Supplementary material for: FLRT2 prevents endothelial cell senescence and vascular aging by regulating the ITGB4/mTORC2/p53 signaling pathway
Source: JCI Insight. 2024 Apr 8;9(7):e172678. doi: 10.1172/jci.insight.172678 (PMC11128196; doi:10.1172/jci.insight.172678)
Supplement: Supplemental data [file jciinsight-9-172678-s046.pdf]

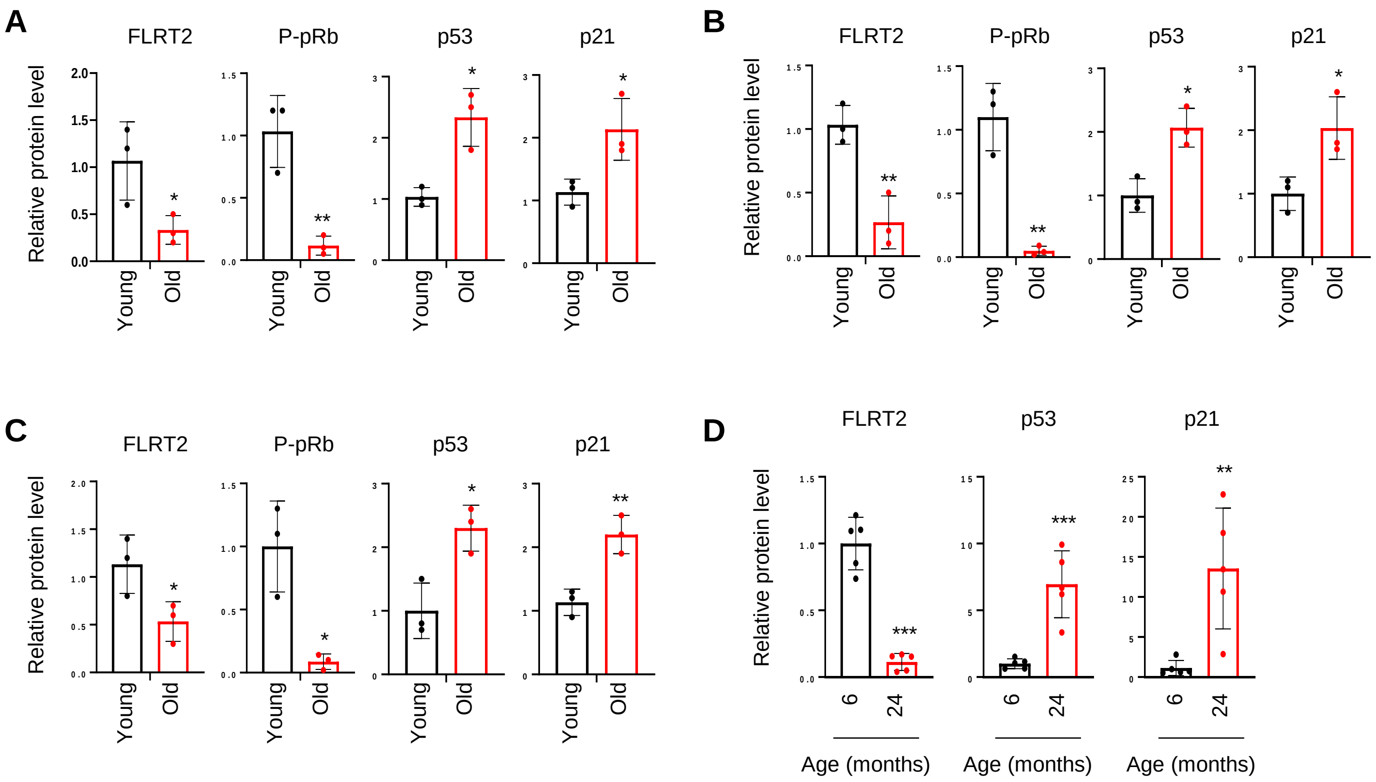


**Supplementary Figure 1. Quantitative analysis of immunoblotting data presented in Fig.1A-D**

**(A-C)** The values represent mean ± SD (n = 3; *P < 0.05; **P < 0.01). **(D)** The values represent mean ± SD (n = 5; **P < 0.01; ***P < 0.001).

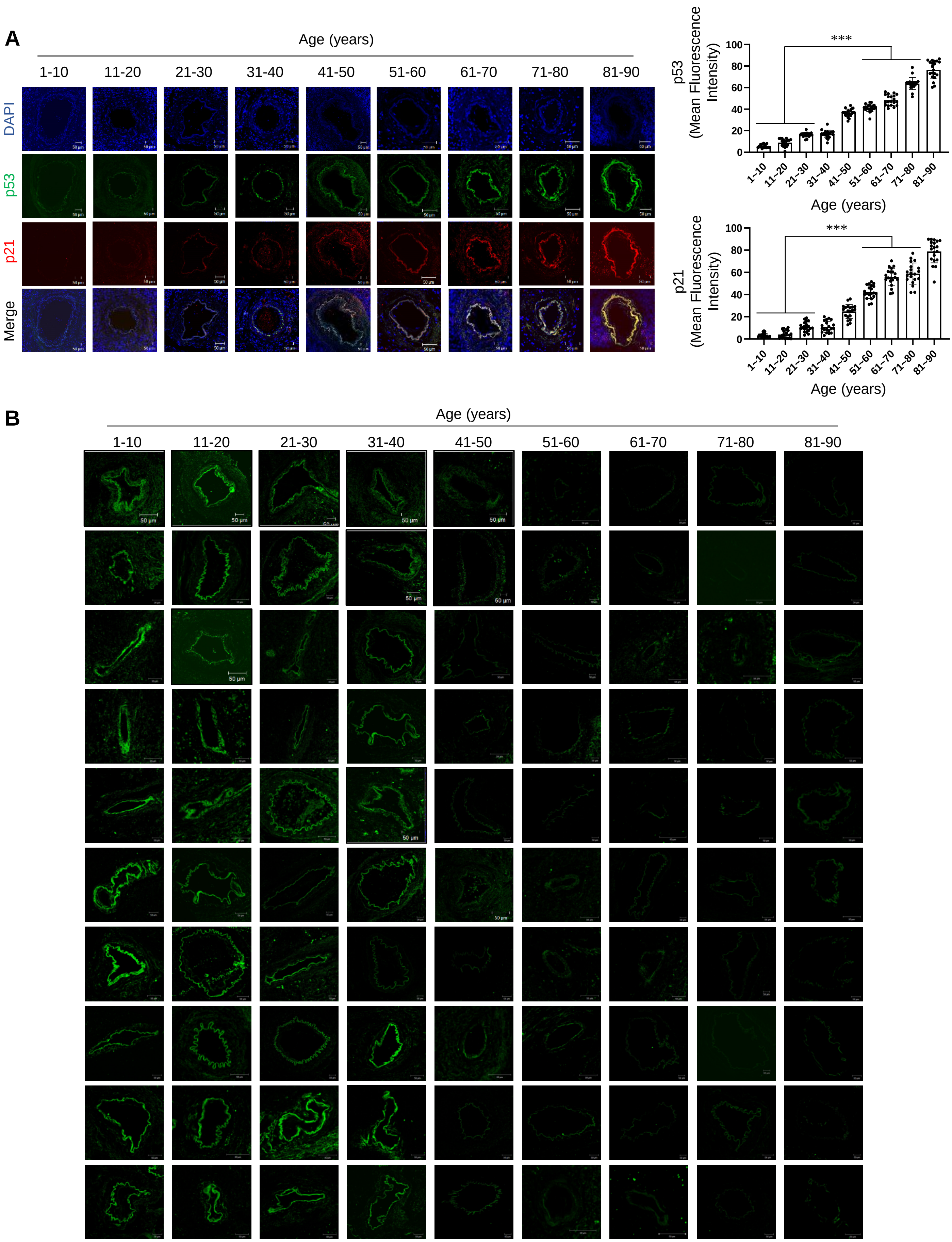

A
Age (years)
1-10
11-20
21-30
31-40
41-50
51-60
61-70
71-80
81-90
DAPI
p53
p21
Merge
p53 (Mean Fluorescence Intensity)
p21 (Mean Fluorescence Intensity)
***
Age (years)
B
Age (years)
50 μm

**Supplementary Figure 2. Upregulation of p53 and p21, and downregulation of FLRT2 in human arterial tissues with age.**

**(A)** Changes in the expression levels of p53 and p21 in human arterial tissues of each age group were detected using immunofluorescence. Scale bar: 50 μm. The values represent mean ± SD (each, n = 20; ***P < 0.001). **(B)** Representative immunohistochemical images of human arterial tissues with age for FLRT2 (each, n = 10). Scale bar: 50 μm.

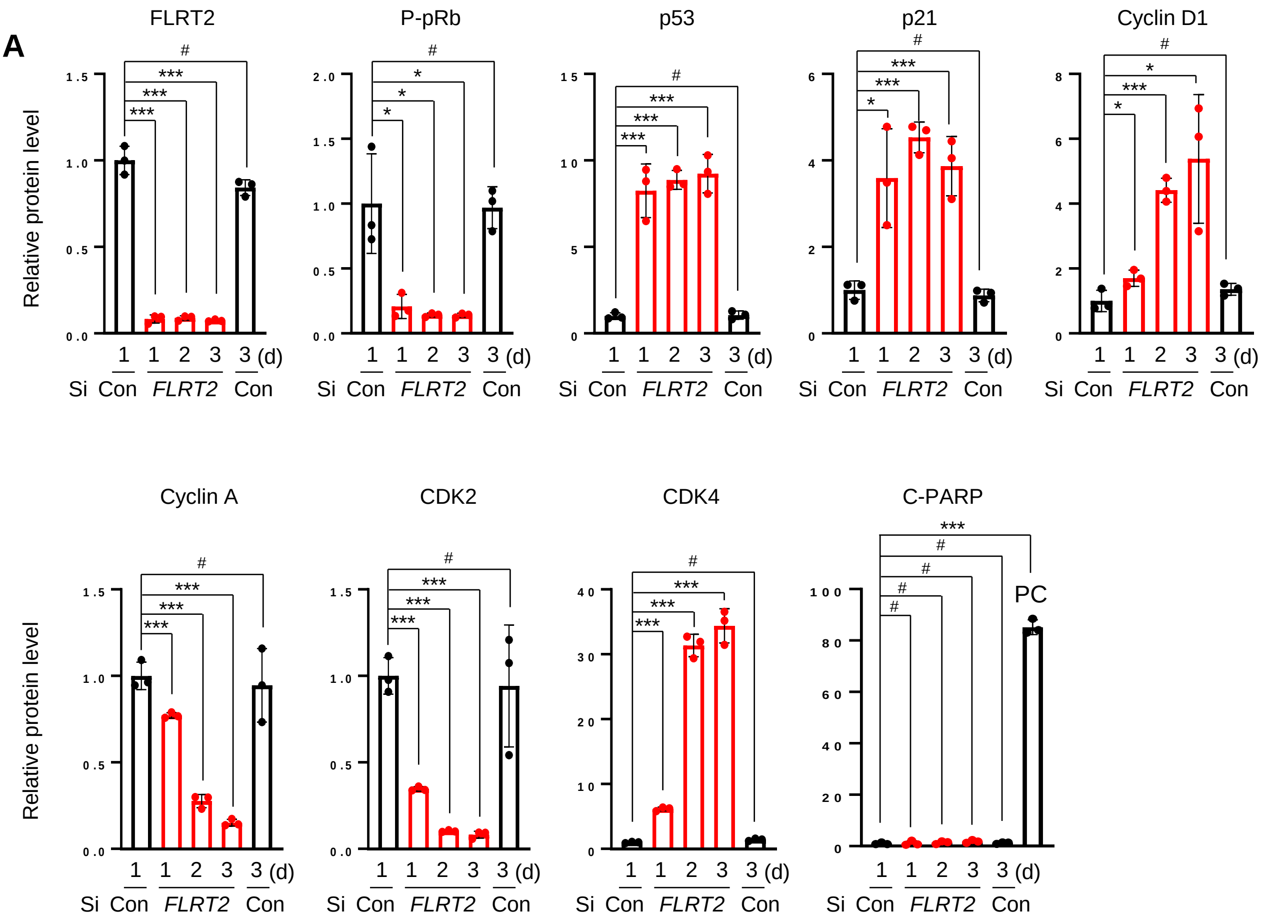


**Supplementary Figure 3. Quantitative analysis of immunoblotting data presented in Fig. 2B**

**(A)** The values represent mean ± SD (n = 3; #P > 0.05; *P < 0.05; ***P < 0.001).

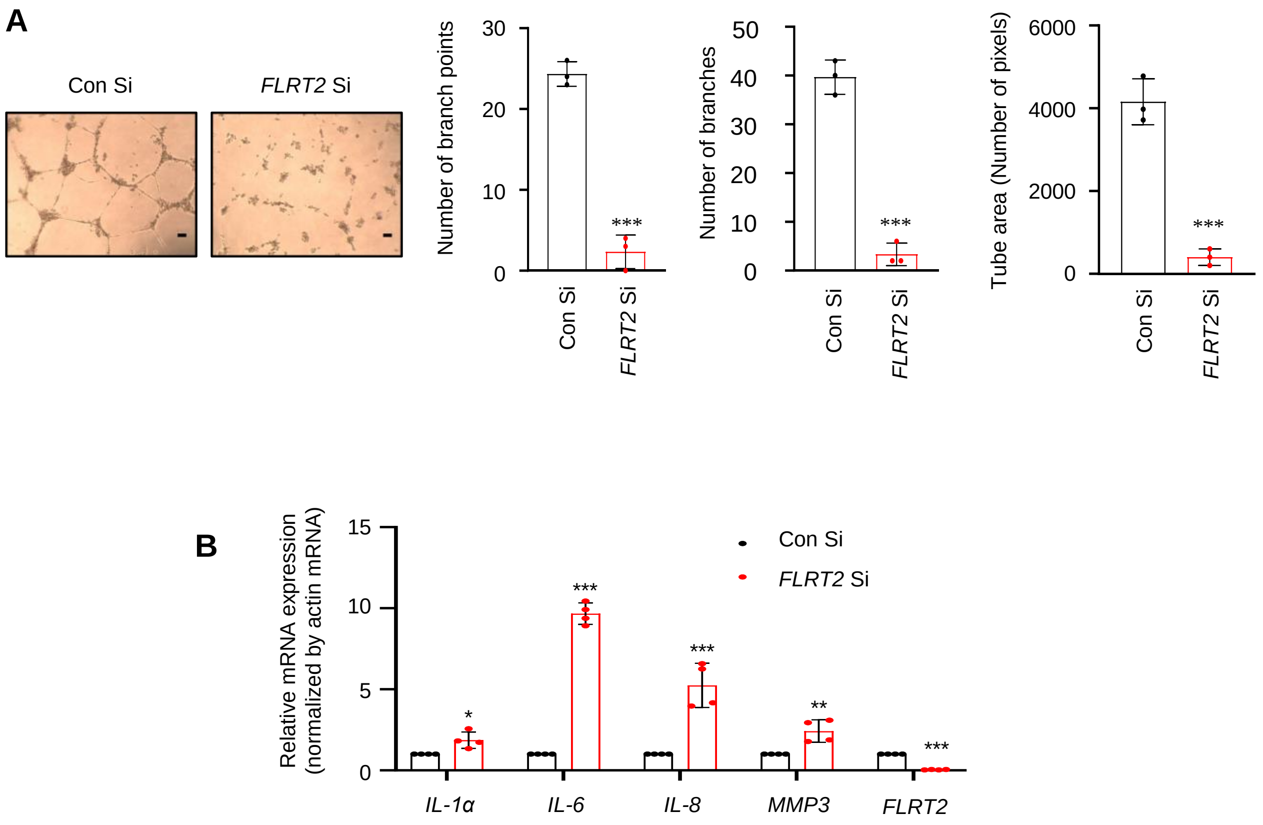


**Supplementary Figure 4. *FLRT2* depletion induces decrease of tube formation ability and increase of expression levels of SASP factors.**

**(A)** Tube formation ability of siRNA-transfected HUVECs was analyzed using the endothelial cell tube formation assay. Scale bar: 100 µm. **(B)** Relative mRNA expression levels of genes related to the senescence-associated secretory phenotype (SASP) in the siRNA-transfected HUVECs were examined using quantitative real-time polymerase chain reaction (qRT-PCR). The values represent mean ± SD (n = 3; #P > 0.05; *P < 0.05; **P < 0.01; ***P < 0.001).

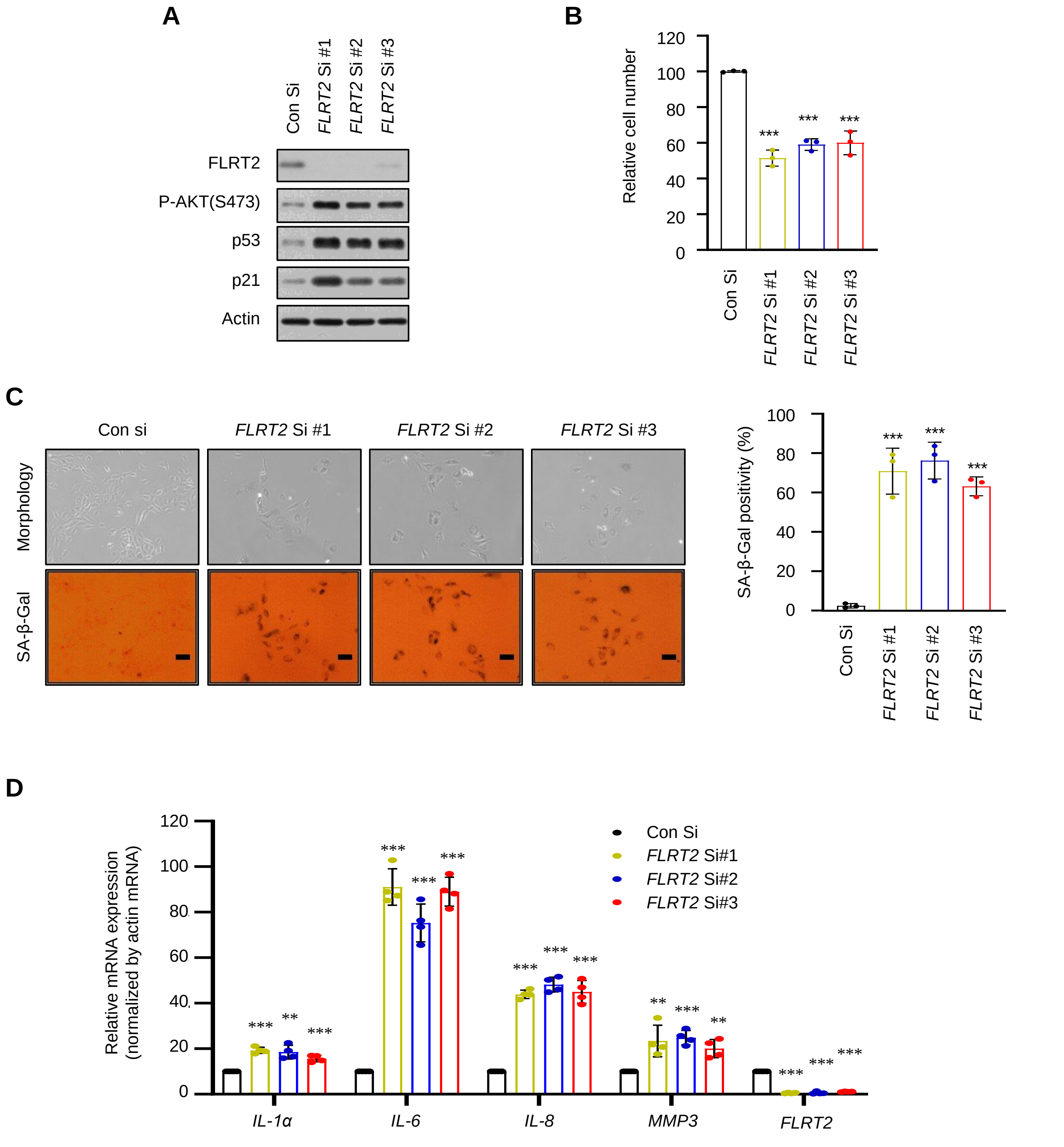


**Supplementary Figure 5. No off-target effects of *FLRT2* siRNAs on endothelial cell senescence.**

**(A-D)** HUVECs were transfected with the indicated siRNAs. **(A)**, Immunoblot assay was performed at day 2 post-transfection. **(B),** Relative cell number, **(C)** SA-β-Gal activity, and **(D)** quantitative real-time polymerase chain reaction (qRT-PCR) analyses were performed at day 3 post-transfection. Scale bar: 10 μm. The values represent mean ± SD (n = 3; **P <0.01; ***P < 0.001).

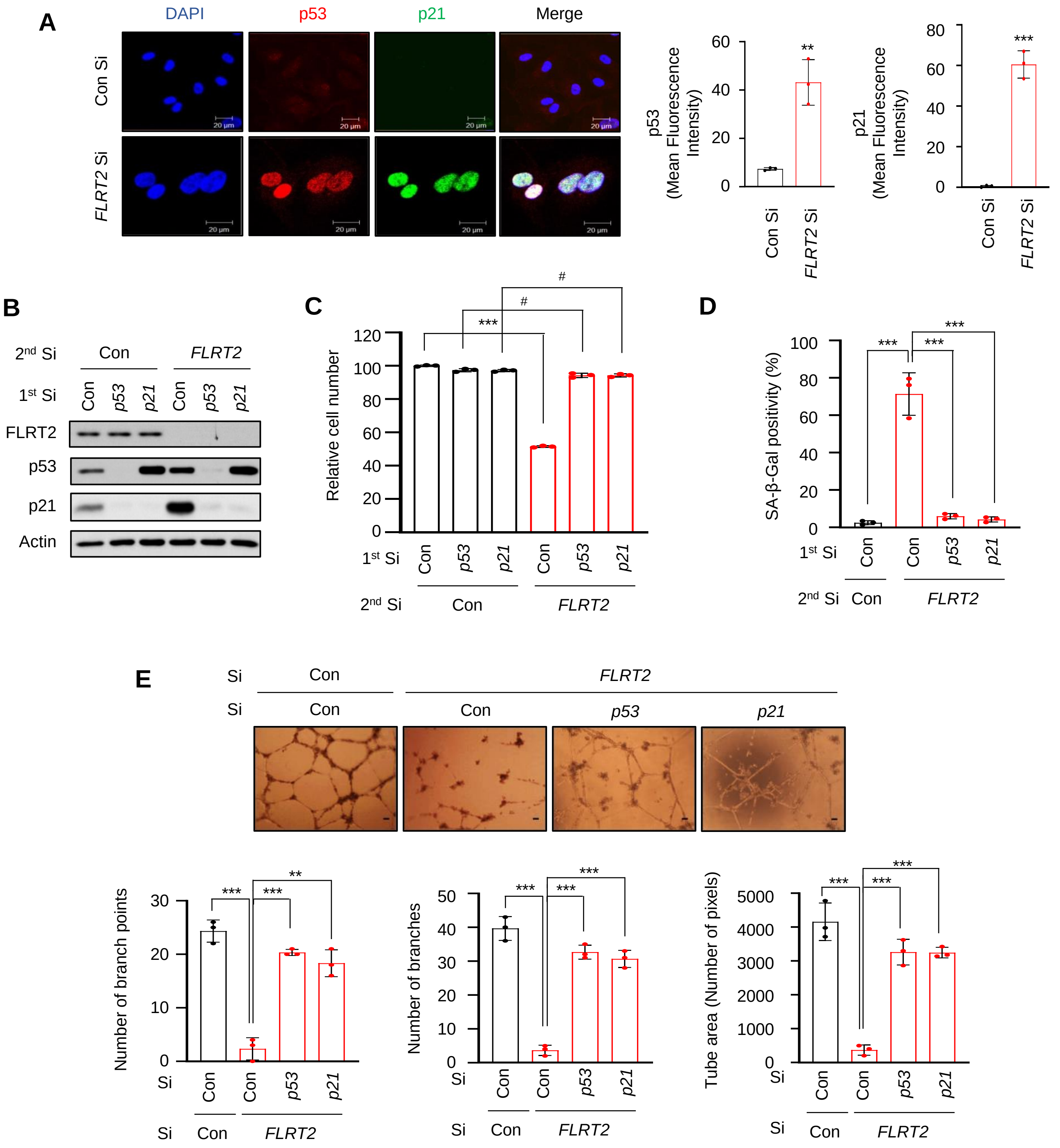


**Supplementary Figure 6. *FLRT2* depletion-induced endothelial cell senescence is mediated by the p53-p21 signaling pathway.**

**(A)** HUVECs were transfected with Con Si or *FLRT2* Si. At day 2 post-transfection, the expression levels of p53 and p21 were examined using immunofluorescence. Quantitative data are shown in the graph. **(B-E)** HUVECs were transfected with Con Si, *p53* Si, or *p21* Si at 6 h before transfection with Con Si or *FLRT2* Si. At day 2 post-transfection, the cells were harvested and subjected to immunoblot assays **(B)**. Relative cell number **(C)**, SA-β-gal activity **(D)**, and endothelial cell tube formation **(E)** were analyzed at day 3 post-transfection. Scale bar: 100 μm. The values represent mean ± SD (n = 3; #P > 0.05; **P <0.01; ***P < 0.001).

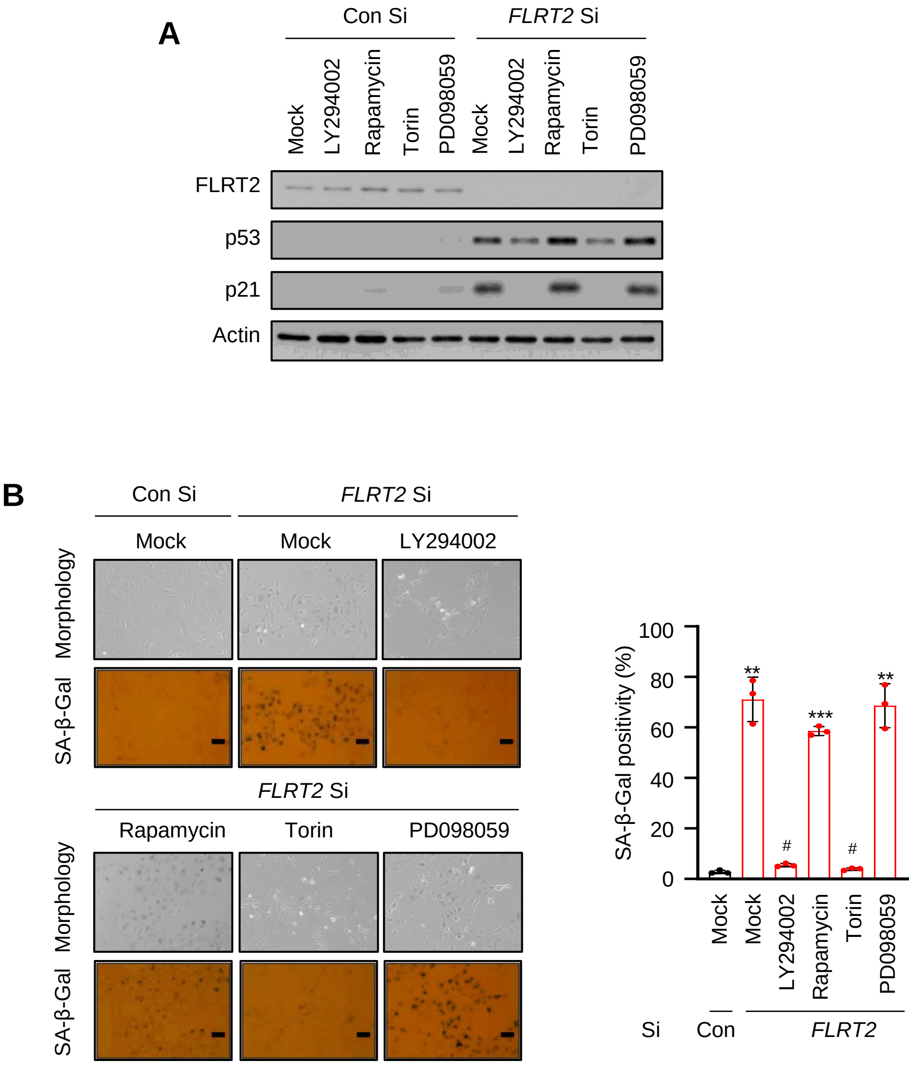


**Supplementary Figure 7. Inhibitors of PI3K and mTORC affect *FLRT2* depletion-mediated endothelial cell senescence.**

**(A, B)** HUVECs were transfected with Con Si or *FLRT2* Si for 24 h and treated with the indicated inhibitors. LY294002, rapamycin, Torin, and PD098059 inhibit PI3K, mTORC1, mTORC1/2, and ERK, respectively. Immunoblot assay was performed at 6 h post-transfection **(A)**. At day 3 post-transfection, SA-β-Gal activity was assessed, and the percentage of senescent cells was quantified. Scale bar: 10 μm **(B)**. **(C-D)** Quantitative analysis of immunoblotting data presented in Fig. 2F-G. The values represent mean ± SD ($n = 3$; #$P > 0.05$; *$P < 0.05$; **$P < 0.01$; ***$P < 0.001$). **(E)** HUVECs were transfected with Con Si or FLRT2 Si. At day 2 post-transfection, cells were fixed and stained with anti-Raptor, anti-Rictor, anti-p53, and anti-p53 (S15) antibodies after permeabilized with Triton X-100. Scale bar, 10 μm. **(F)** HUVECs were transfected with Con Si or FLRT2 Si. At day 2 post-transfection, cell lysates were subjected to IP with anti-p53 antibody, and performed immunoblot assays with the indicated antibodies.

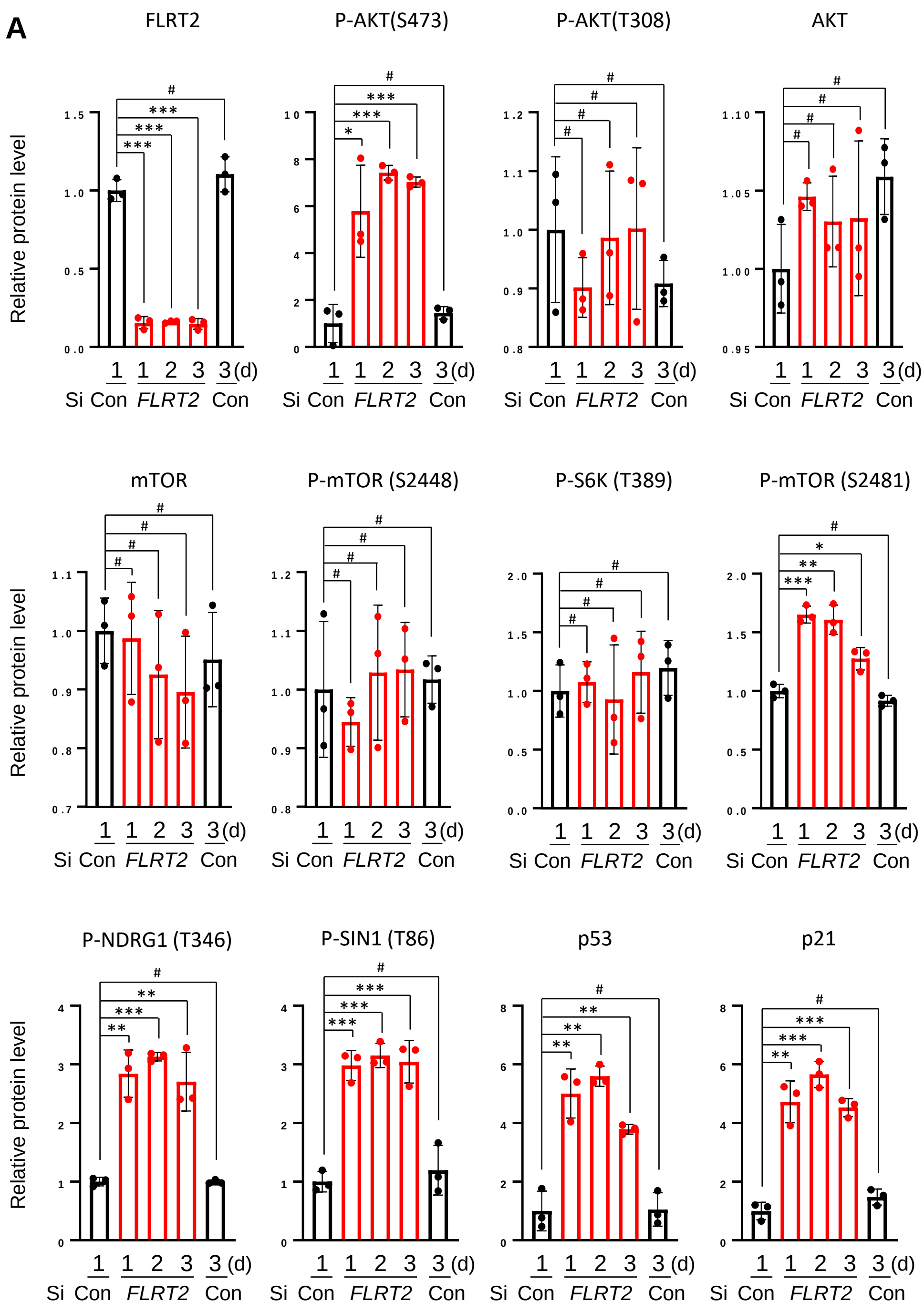


**Supplementary Figure 8. Continued**

**B**

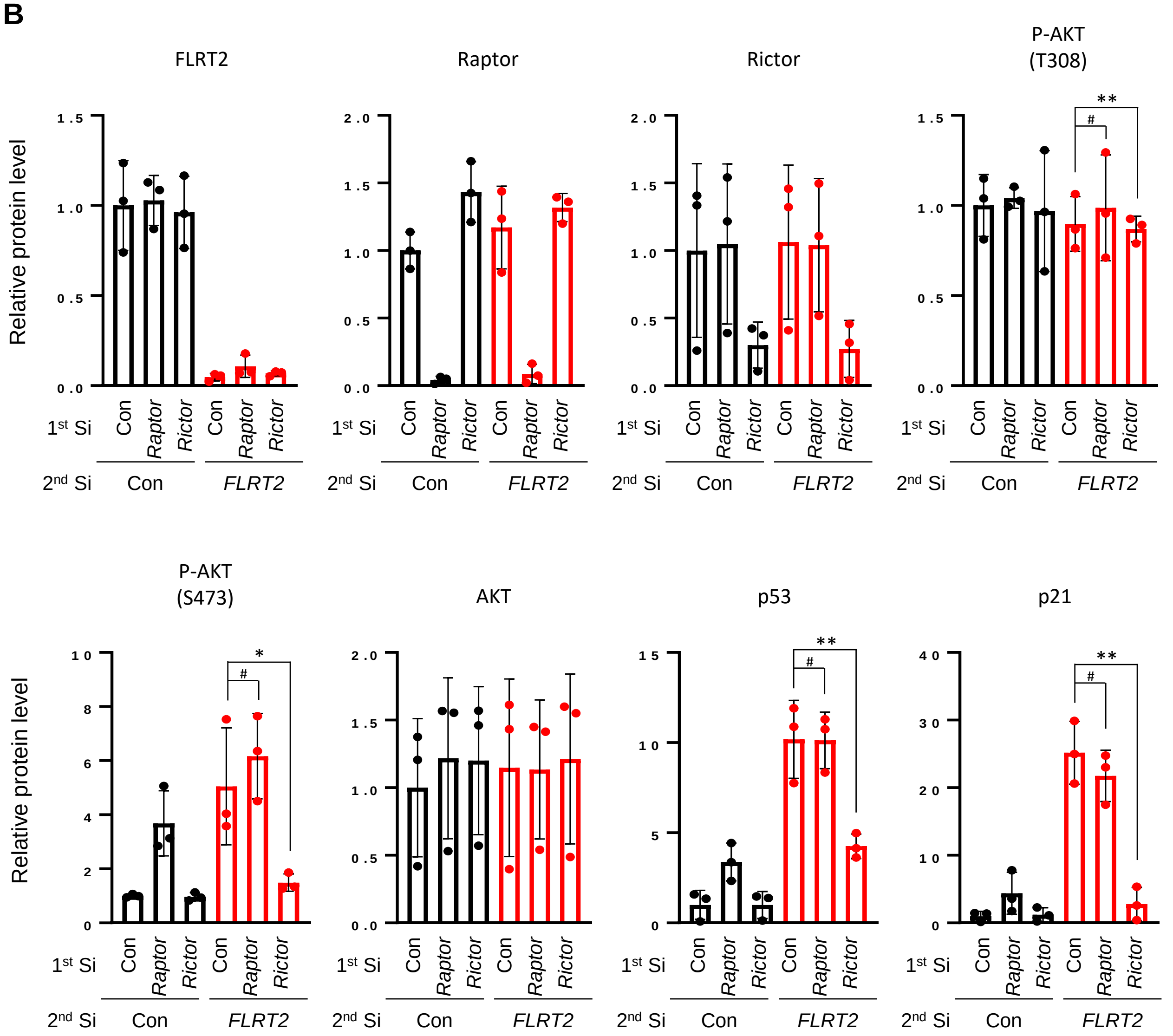


**Supplementary Figure 8. Quantitative analysis of immunoblotting data presented in Fig. 2F-G**

**(A-B)** The values represent mean ± SD (n = 3; #P > 0.05; *P < 0.05; **P < 0.01; ***P < 0.001).

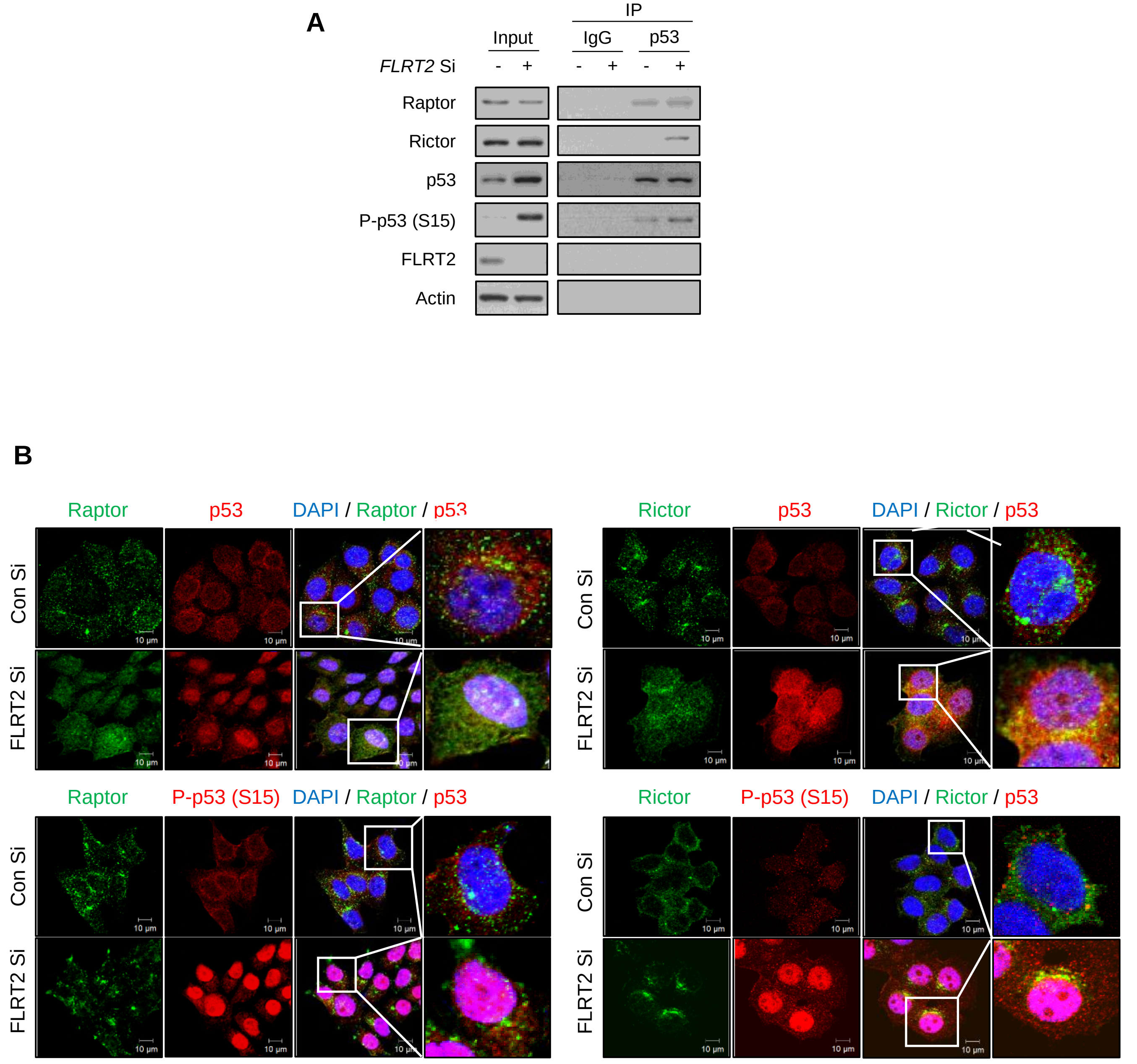


**Supplementary Figure 9. Localization of Raptor, Rictor, and p53 in FLRT2-depleted HUVECs.**

**(A)** HUVECs were transfected with Con Si or FLRT2 Si. At day 2 post-transfection, cell lysates were subjected to IP with anti-p53 antibody and performed immunoblot assays with the indicated antibodies. **(B)** HUVECs were transfected with Con Si or FLRT2 Si. At day 2 post-transfection, cells were fixed and stained with anti-Raptor, anti-Rictor, anti-p53, and anti-p53 (S15) antibodies after permeabilized with Triton X-100. Scale bar, 10 µm.

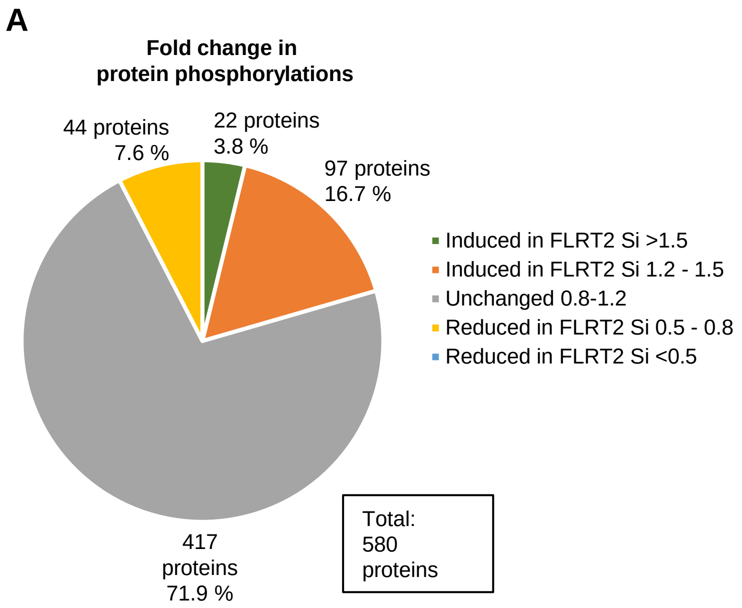


**B**

| Candidates | Fold change |
|---|---|
| P-FGFR1 (Tyr766) | 1.455 |
| P-VEGFR2 (Tyr1059) | 1.281 |
| P-EGFR (Tyr1016) | 1.23 |
| P-ITGB4 (Tyr1510) | 1.219 |

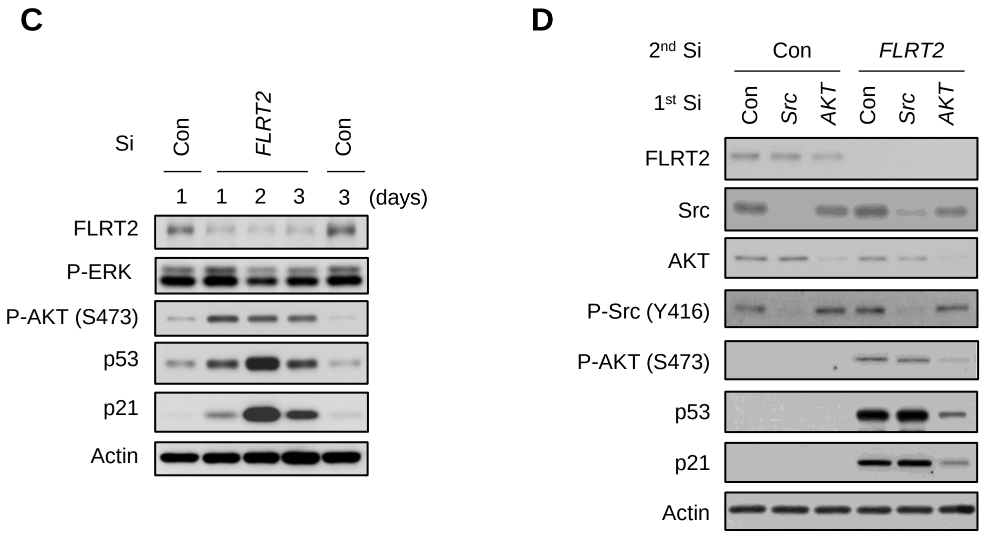


**Supplementary Figure 10. Analysis of protein phosphorylation changes in FLRT2-depleted HUVECs.**

**(A-B)** The Phospho Explorer Antibody Array was used to screen total cell lysates from Con Si or *FLRT2* Si-transfected HUVECs cultures. The numbers of legends in the pie diagram indicate the fold change in protein phosphorylation of *FLRT2* Si-transfected cells, i.e. induced (green, orange), reduced (yellow), and unchanged (gray) **(A).** The data shows the fold change of the indicated phosphoproteins 2 days after transfection with Con Si or *FLRT2* Si **(B). (C)** HUVECs were transfected with Con Si or FLRT2 Si, harvested at the indicated time points post-transfection, and subjected to immunoblotting. **(D)** HUVECs were transfected with Con Si, Src Si, or AKT Si (1st transfection) 6 h before transfection with Con Si or FLRT2 Si (2nd transfection). At day 2 post-transfection, immunoblot assay was performed.

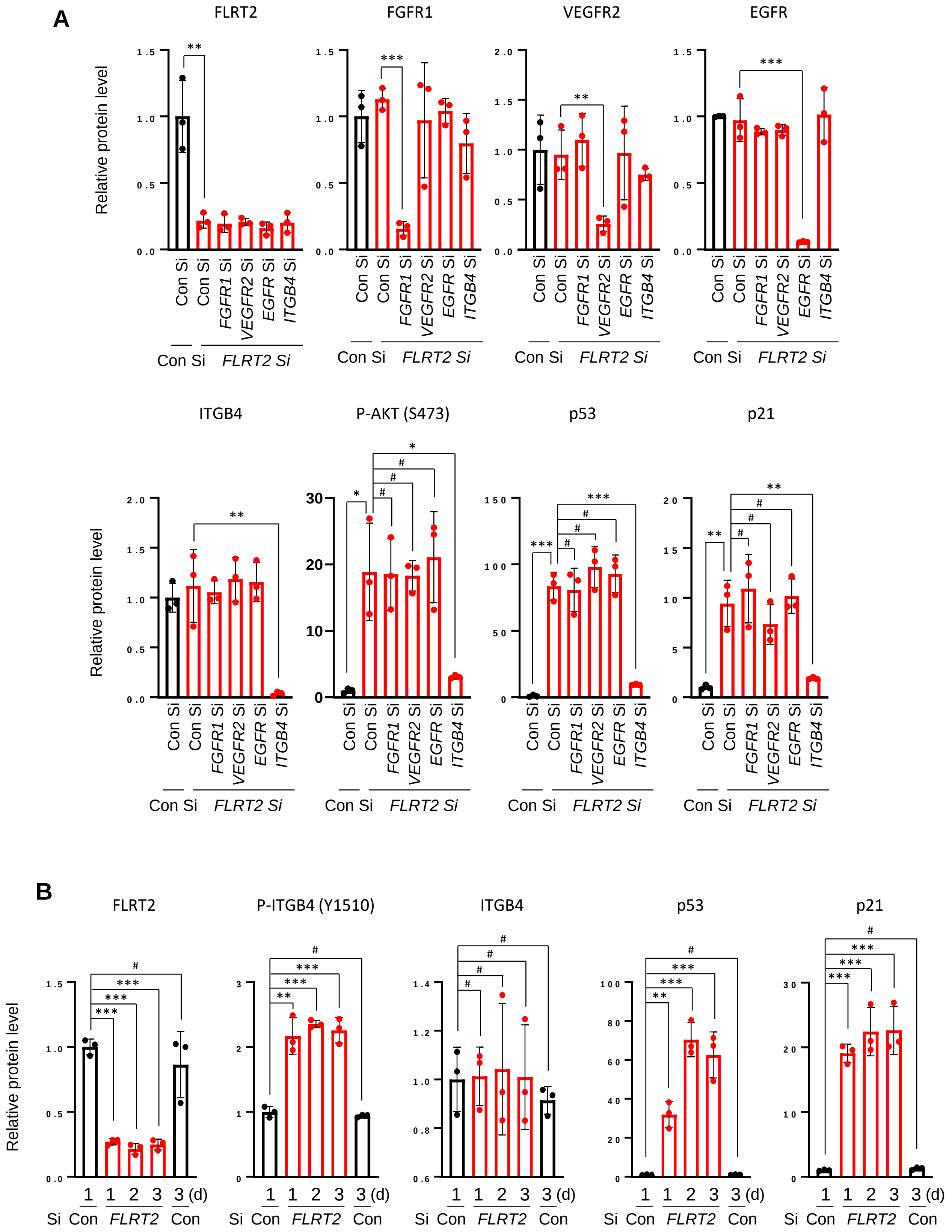
A
FLRT2
FGFR1
VEGFR2
EGFR
ITGB4
P-AKT (S473)
p53
p21
Relative protein level
Con Si
FGFR1 Si
VEGFR2 Si
EGFR Si
ITGB4 Si
FLRT2 Si
B
FLRT2
P-ITGB4 (Y1510)
ITGB4
p53
p21
Relative protein level
Si
Con
FLRT2
(d)

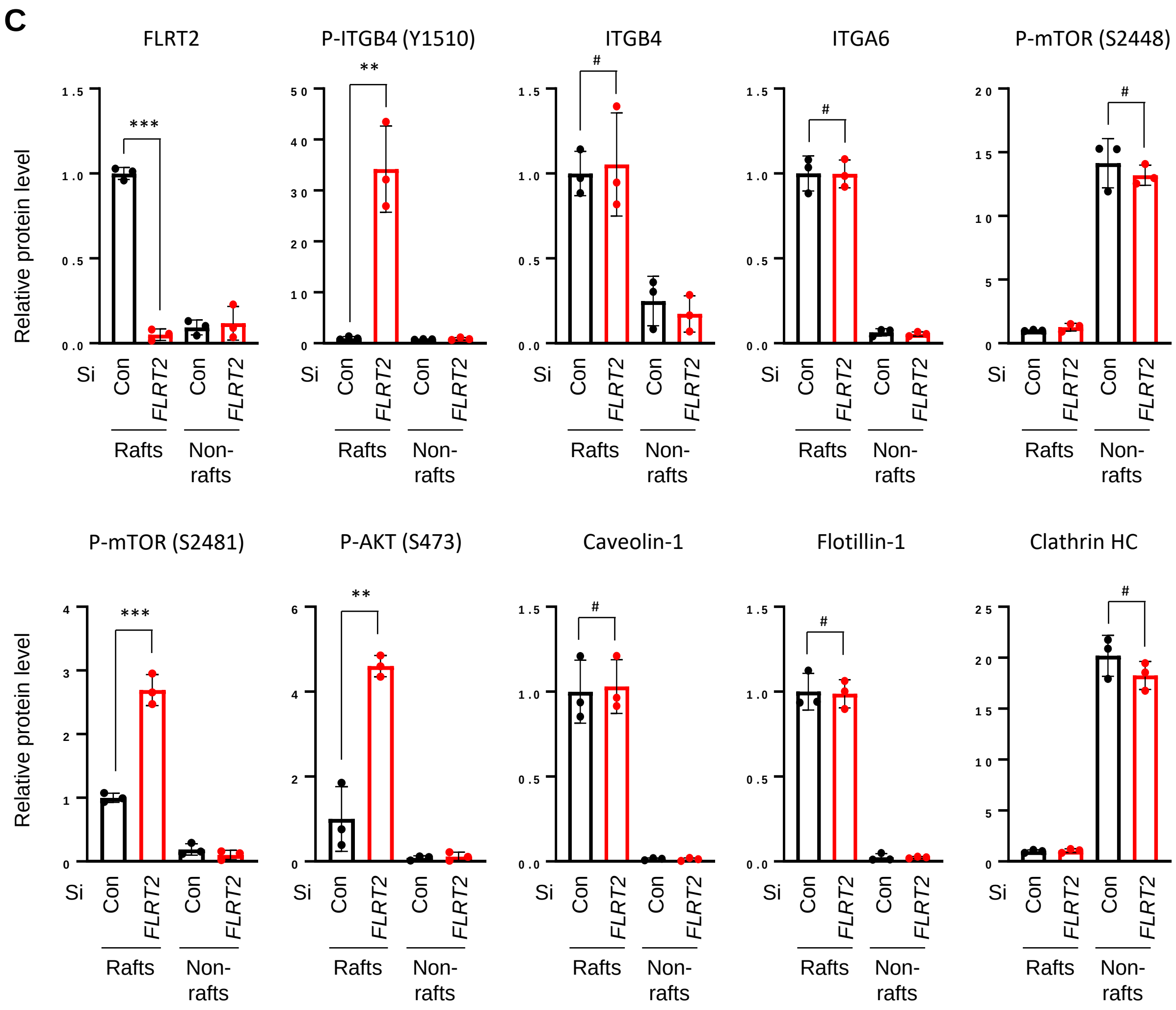


**Supplementary Figure 11. Quantitative analysis of immunoblotting data presented in Fig. 3A, 3B, and 3G.**

**(A-C)** The values represent mean ± SD (n = 3; #P > 0.05; *P < 0.05; **P < 0.01; ***P < 0.001).

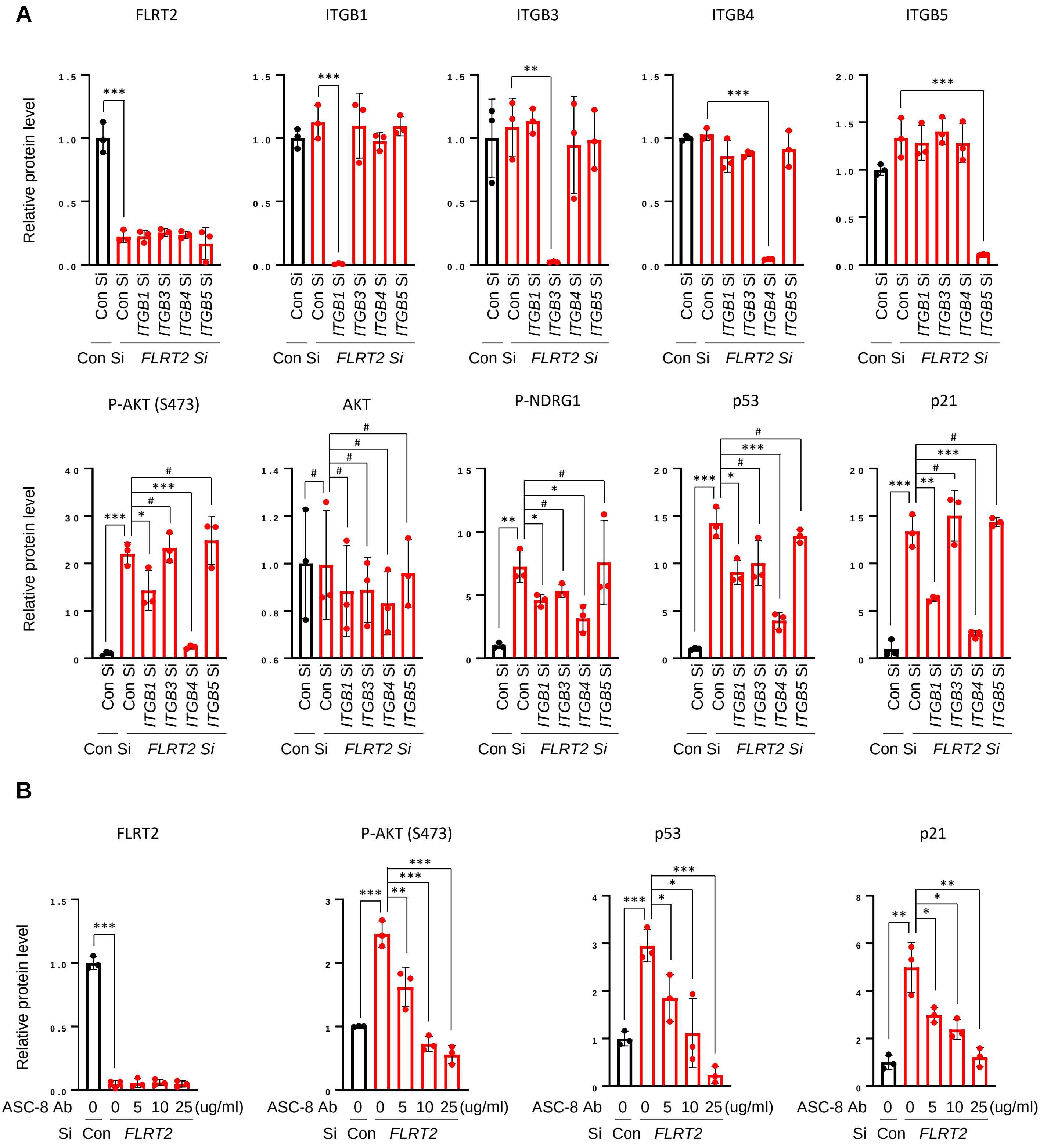


**Supplementary Figure 12. Continued**

C

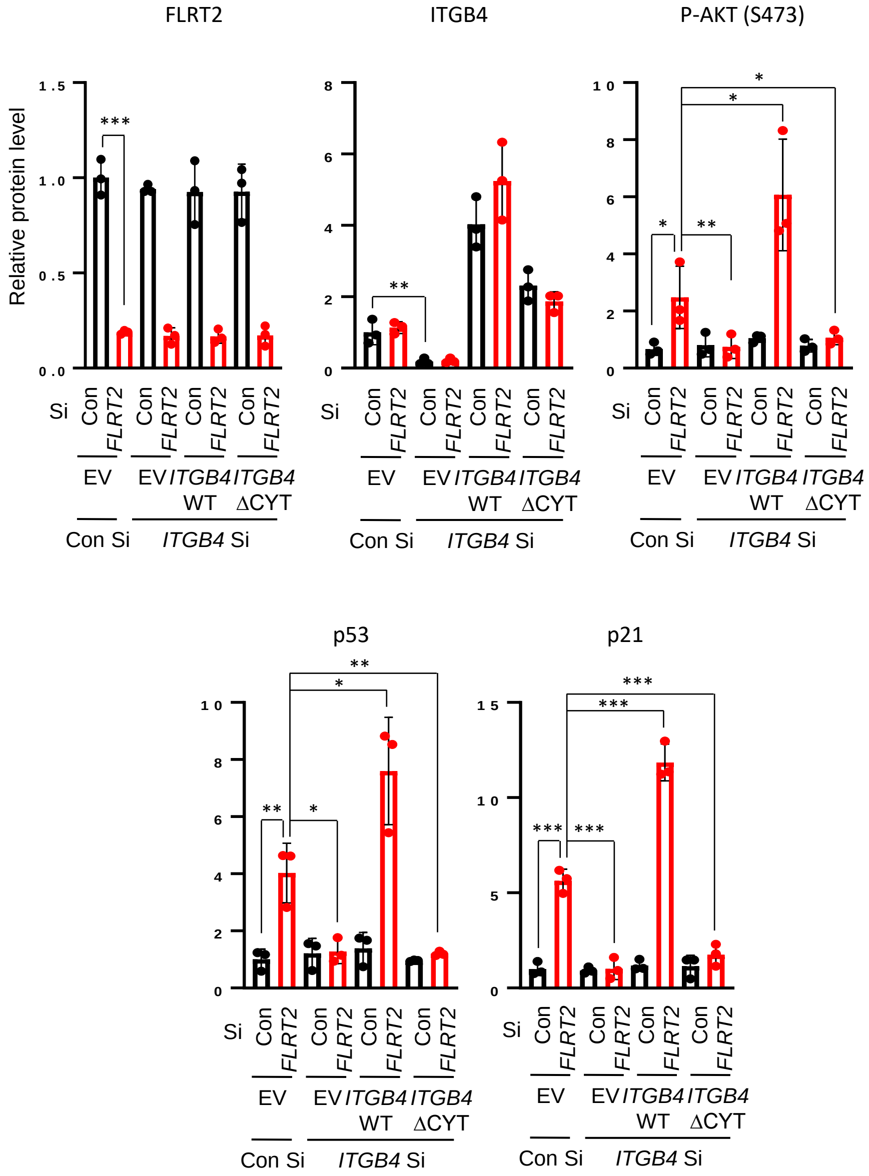


**Supplementary Figure 12. Quantitative analysis of immunoblotting data presented in Fig. 4A, 4D and 4F. (A-C)** The values represent mean ± SD (n = 3; #P > 0.05; *P < 0.05; **P < 0.01; ***P < 0.001).

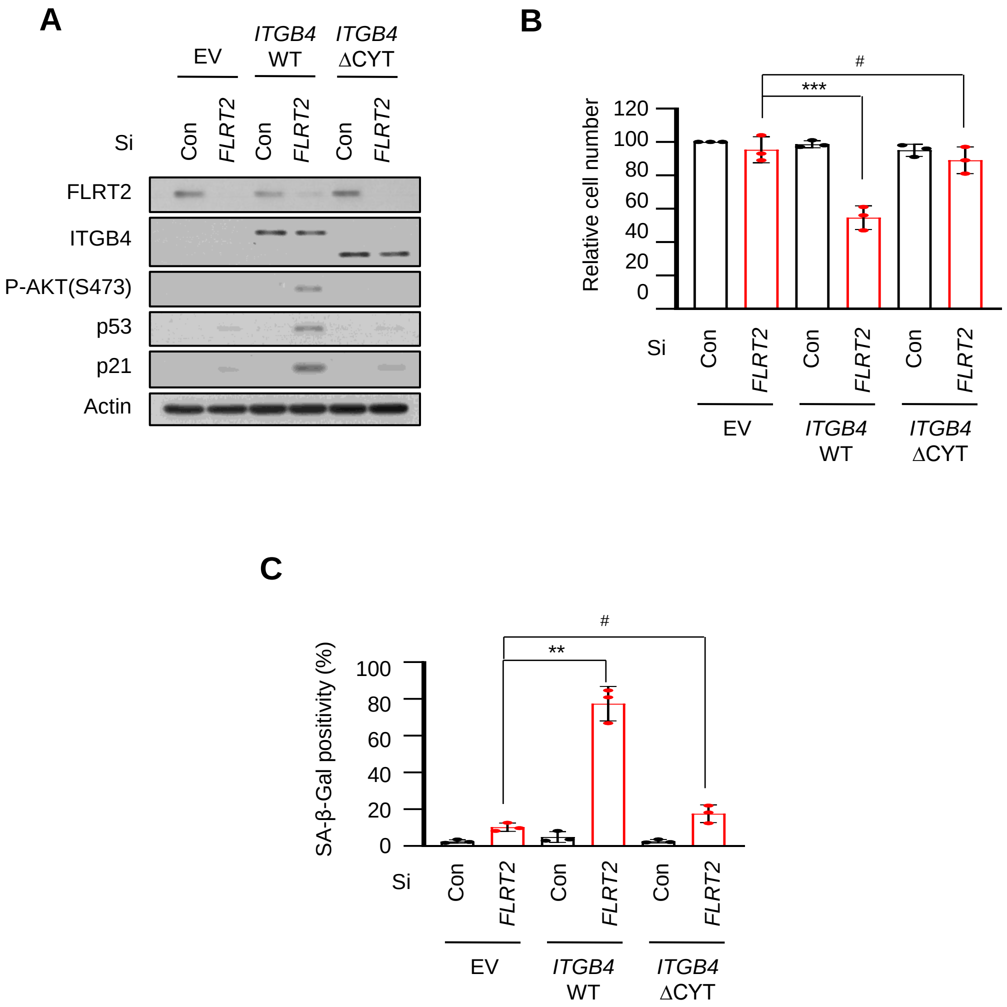


**Supplementary Figure 13. The phosphorylation domain of integrin β4 involves in *FLRT2* depletion-induced senescence in human aortic endothelial cells (HAECs).**

**(A-C)** Quantitative analysis of immunoblotting data presented in Fig. 4A, 4D and 4F. The values represent mean ± SD (n = 3; #P > 0.05; *P < 0.05; **P < 0.01; ***P < 0.001). **(D-F)** HAEC were transfected with empty vector (EV) or a vector encoding wild-type *ITGB4* (WT) or a truncated mutant of *ITGB4* lacking the residues downstream of amino acid 1355 (ΔCYT), followed by transfection with Con Si or *FLRT2* Si. **(D)** Cell lysates were subjected to immunoblotting. Relative cell number **(E)** and SA-β-Gal activity **(F)** were measured at day 3 post-transfection. The values represent mean ± SD (n = 3; #P > 0.05; **P <0.01; ***P < 0.001).

**A**

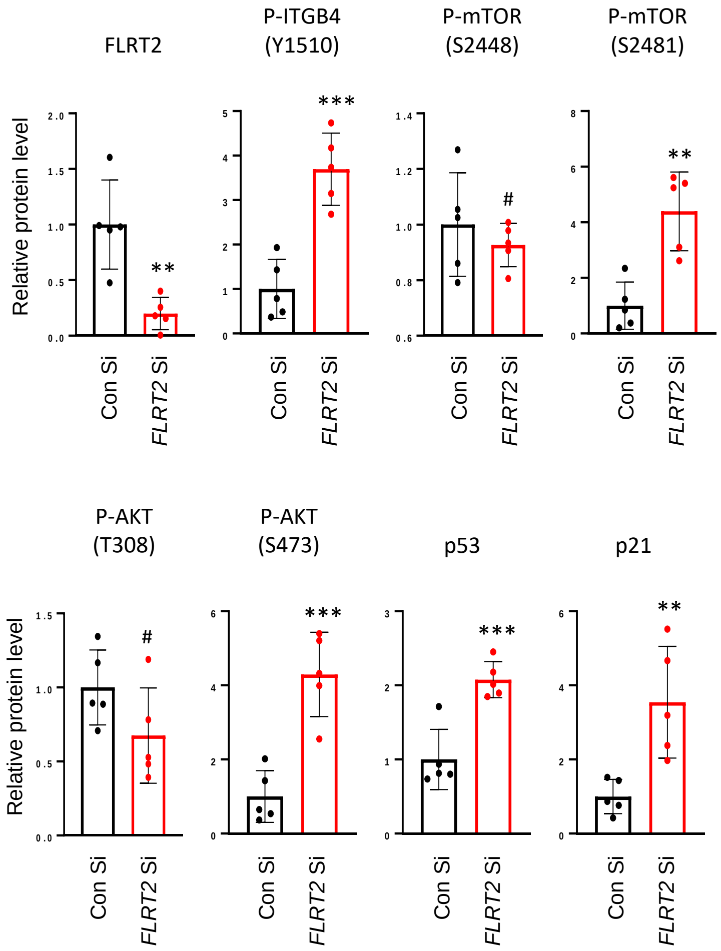


**Supplementary Figure 14. Quantitative analysis of immunoblotting data presented in Fig. 5A.**

(A). The values represent mean ± SD (n = 3; #P > 0.05; **P < 0.01; ***P < 0.001).

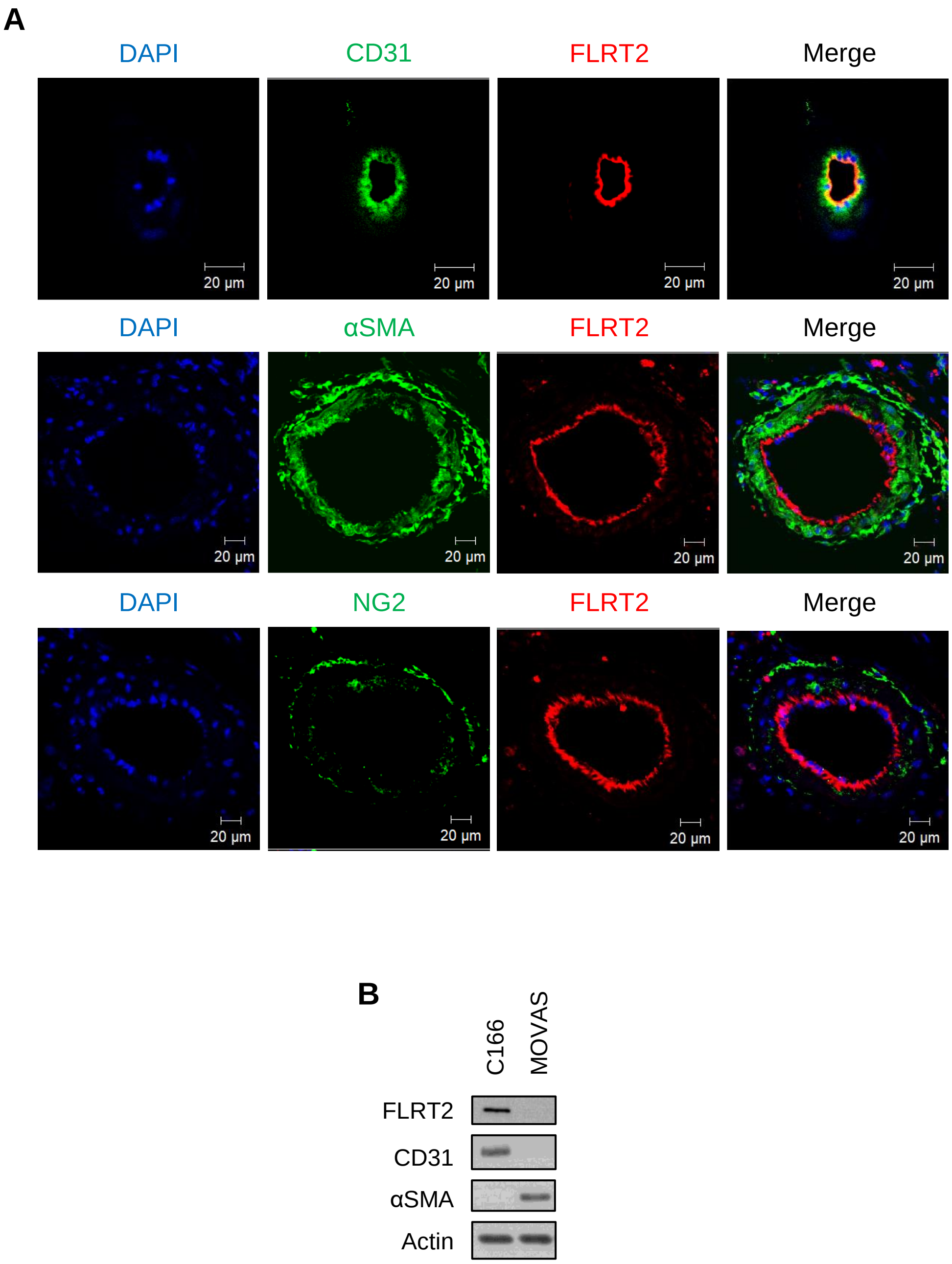


**Supplementary Figure 15. Localization and expression of FLRT2 in mouse aorta and mouse cell lines.**

**(A)** Representative images of immunofluorescence staining of CD31, αSMA, NG2, and FLRT2 in aortic cross sections of mice. **(B)** C166 and MOVAS cells were subjected to immunoblot assays with the indicated antibodies.

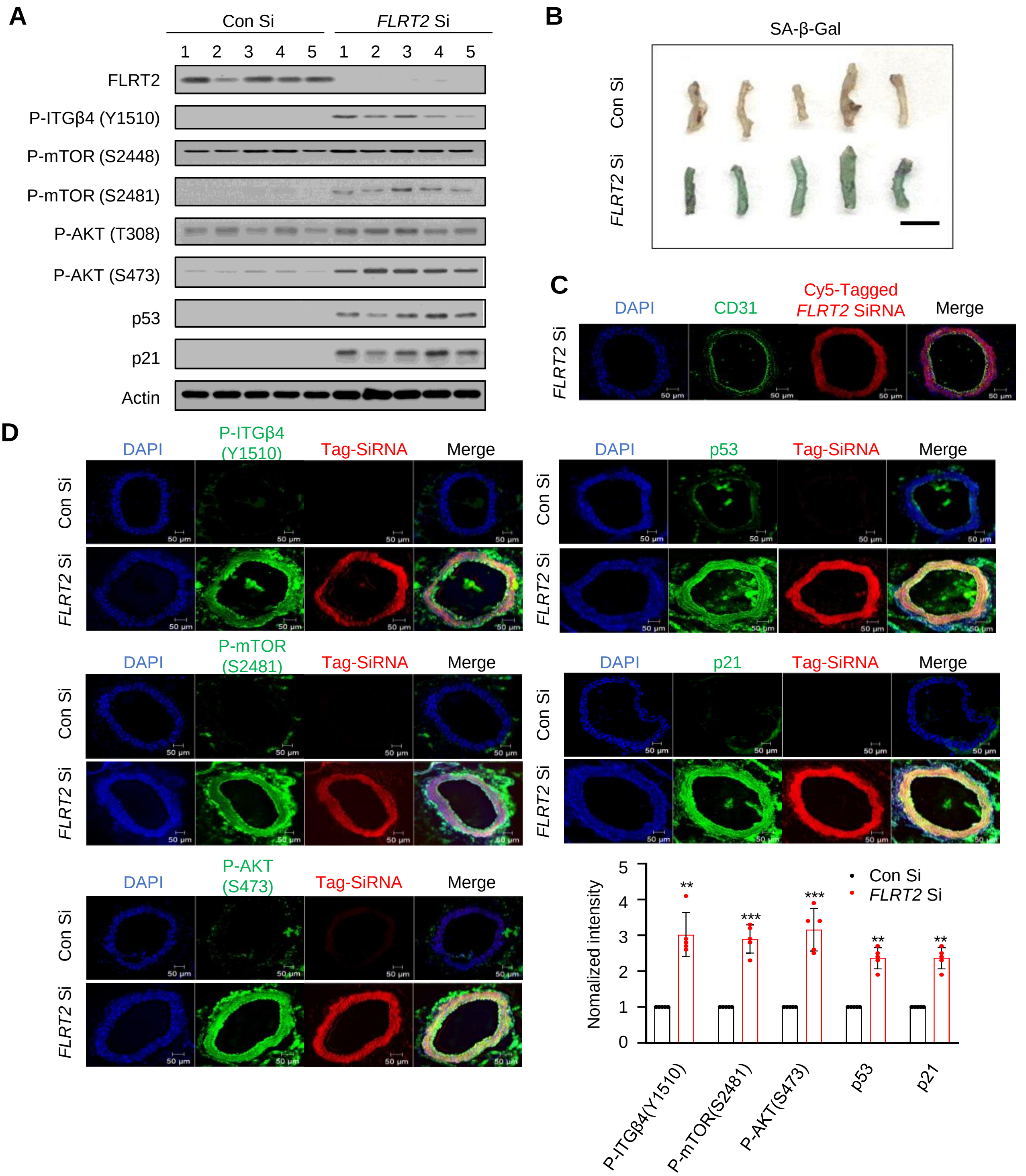


**Supplementary Figure 16.** ***In vivo*** **knockdown of** ***FLRT2*** **promotes cell senescence in veins of mice.**

**(A)** Representative immunoblot images for venous lysates of mice injected intravenously with Con Si or *FLRT2* Si (n = 5). **(B)** Image of SA-β-Gal-stained veins of mice. Scale bar: 2 mm. **(C)** Fluorescence microscopies of venous cross sections obtained from mice injected intravenously with Cy5–tagged *FLRT2* Si. Nuclei are stained with DAPI (blue). Red and green signals show Cy5–tagged *FLRT2* Si and the endothelial marker, CD31, respectively. Merged image indicates siRNA uptake by the endothelium. **(D)** Immunostaining for P-ITGB4 (Y1510), P-mTOR (S2481), P-AKT (S473), p53, and p21 in veins of mice injected with the indicated siRNAs. The values represent mean ± SD (n = 5; **P <0.01; ***P < 0.001).

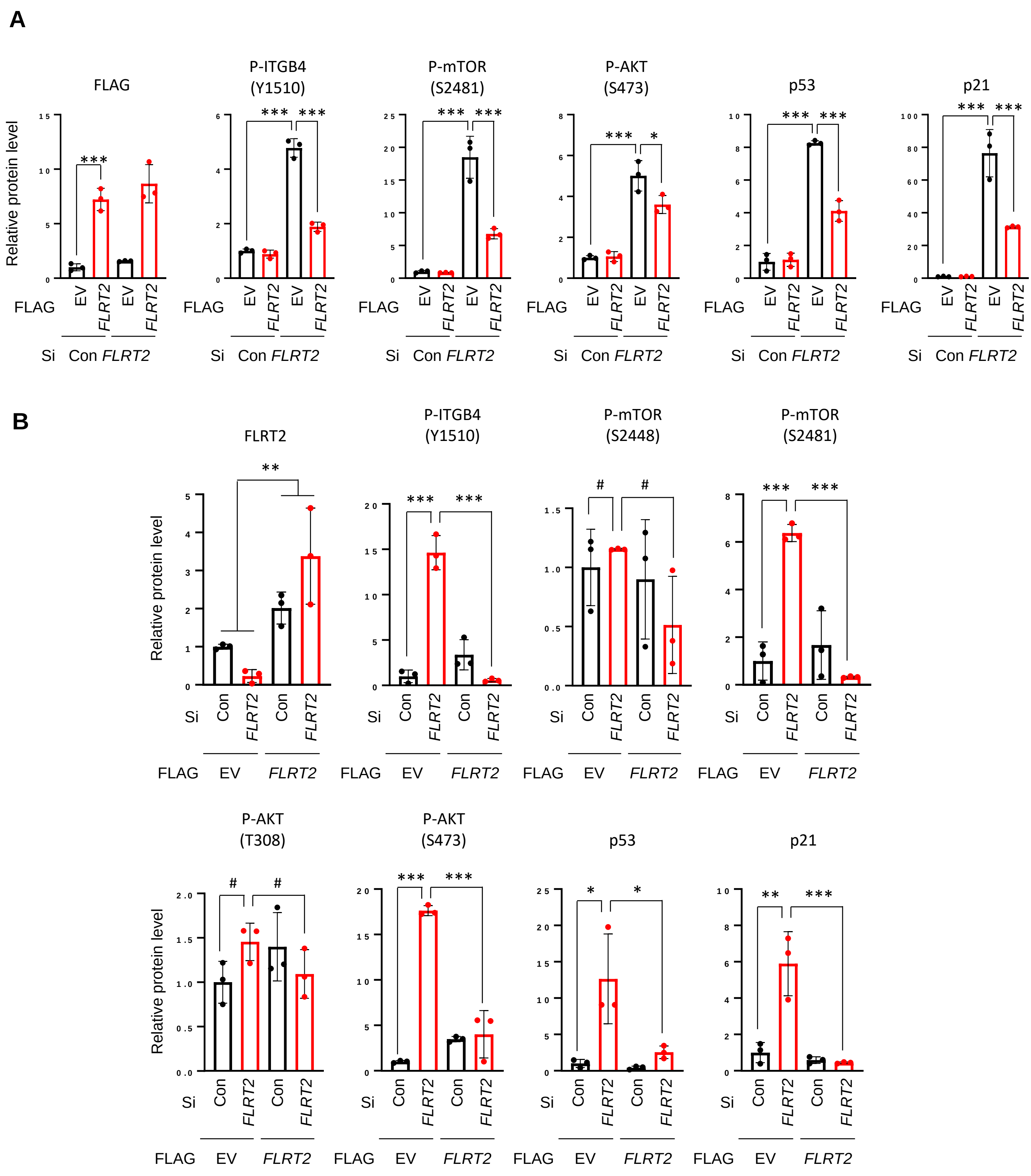


**Supplementary Figure 17. Quantitative analysis of immunoblotting data presented in Fig. 6A and 6D.**

**(A-B)**The values represent mean ± SD (n = 3; #P > 0.05; *P < 0.05; **P < 0.01; ***P < 0.001).
