# Supplementary material for: FLRT2 prevents endothelial cell senescence and vascular aging by regulating the ITGB4/mTORC2/p53 signaling pathway
Source: JCI Insight. 2024 Apr 8;9(7):e172678. doi: 10.1172/jci.insight.172678 (PMC11128196; doi:10.1172/jci.insight.172678)

Full unedited gel for Figure 1.

Fig. 1A

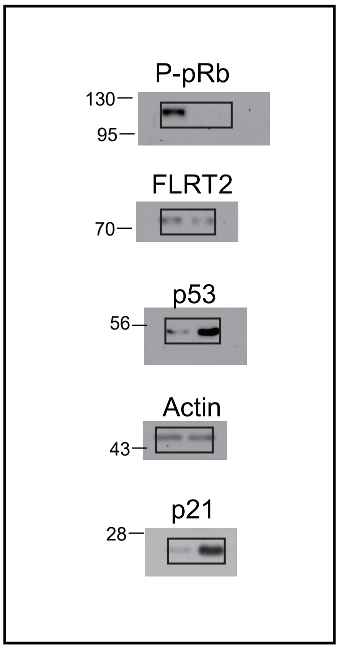

Fig. 1B

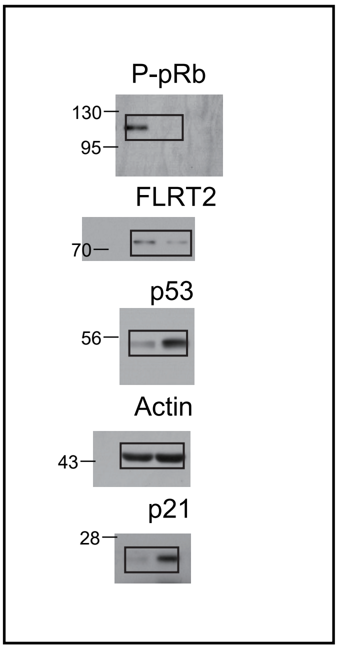

Fig. 1C

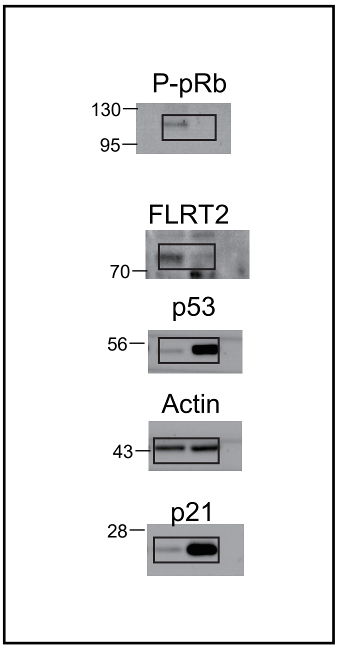

Fig. 1D

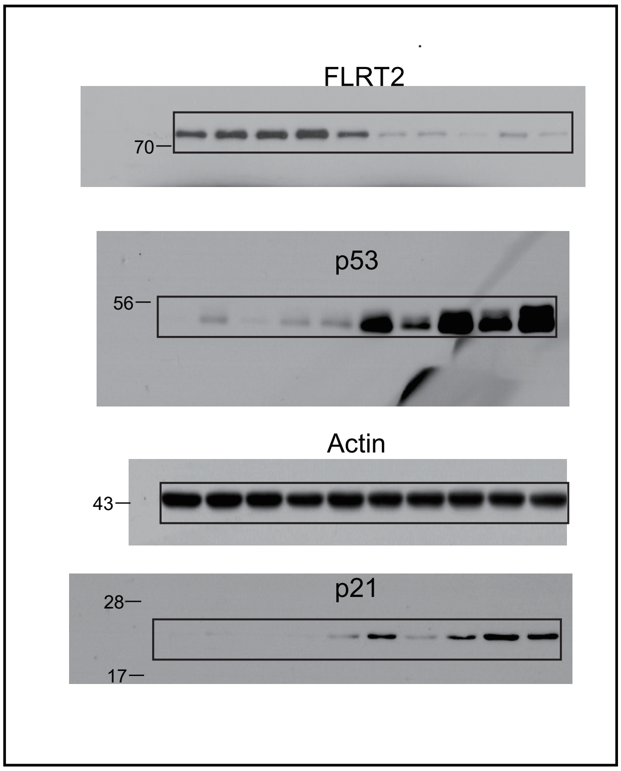

Full unedited gel for Figure 2.

Fig. 2B

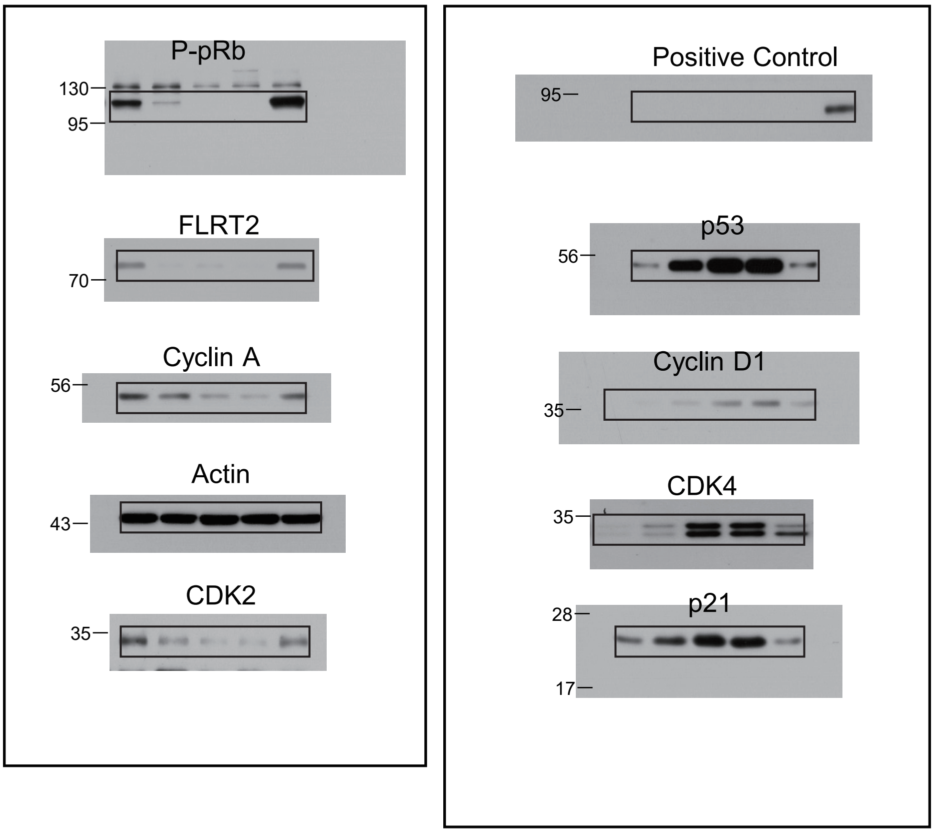

Full unedited gel for Figure 2.

Fig. 2F

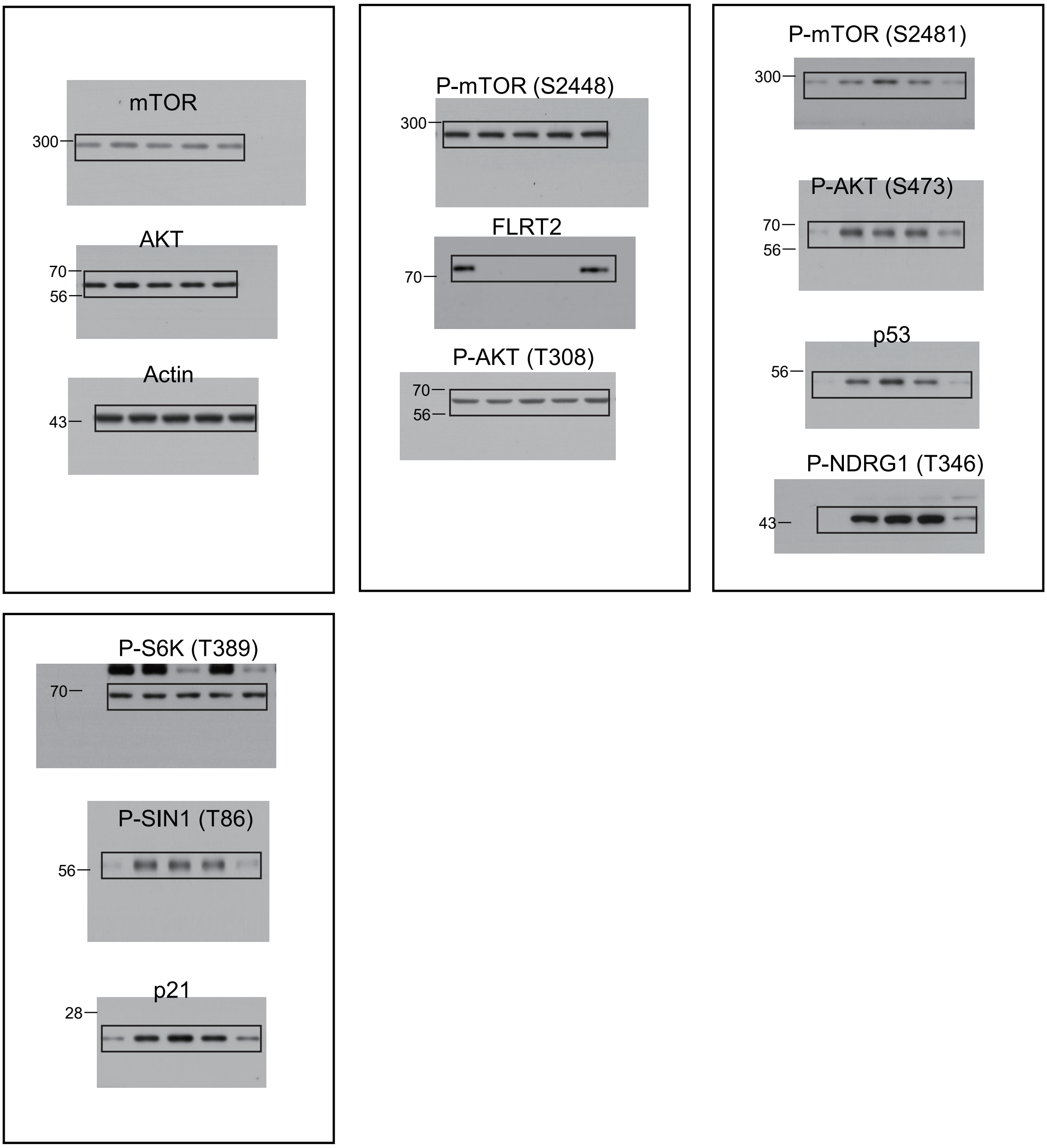

Full unedited gel for Figure 2.

Fig. 2G

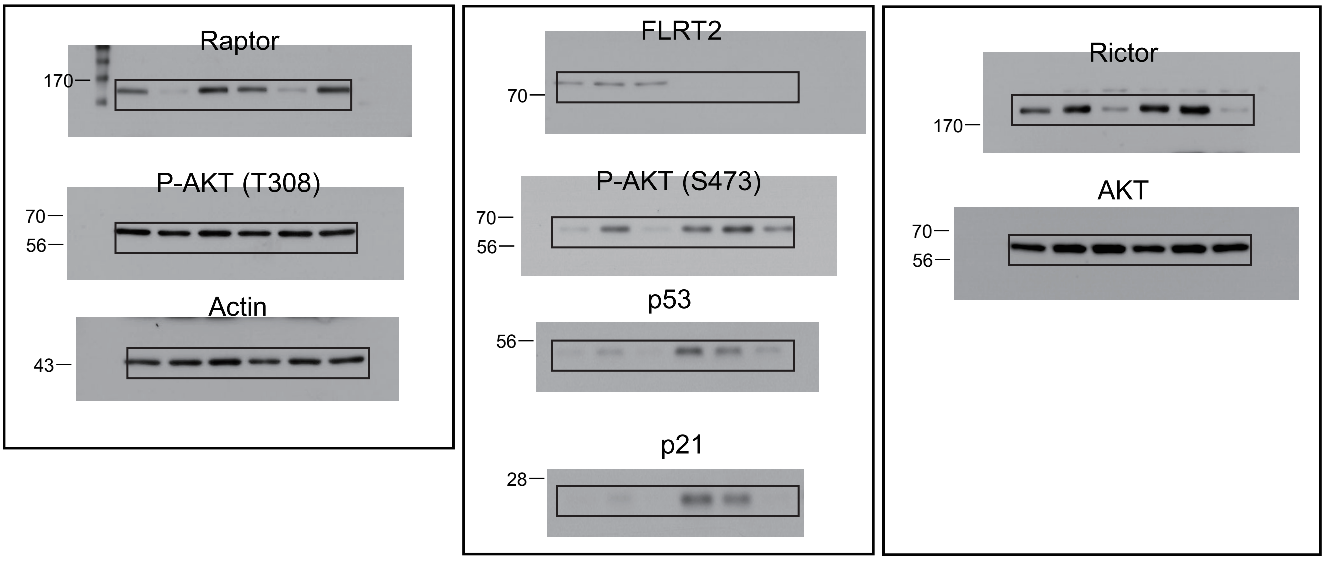

Fig. 2I

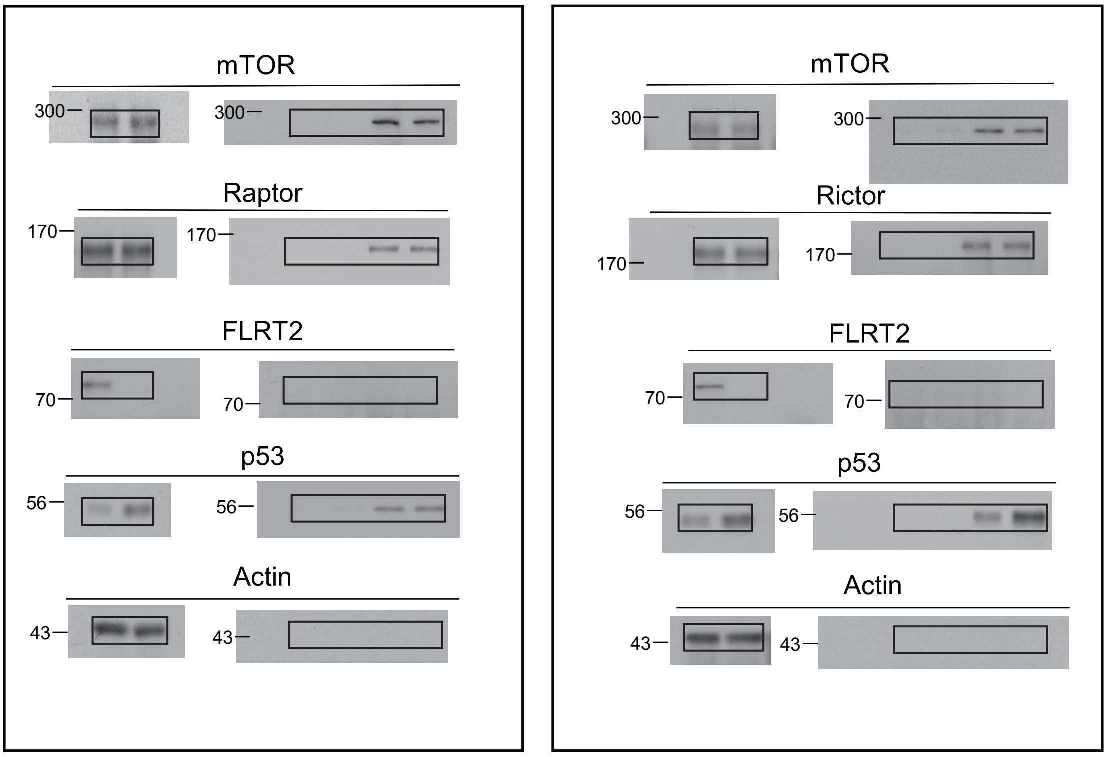

Full unedited gel for Figure 3.

Fig. 3A

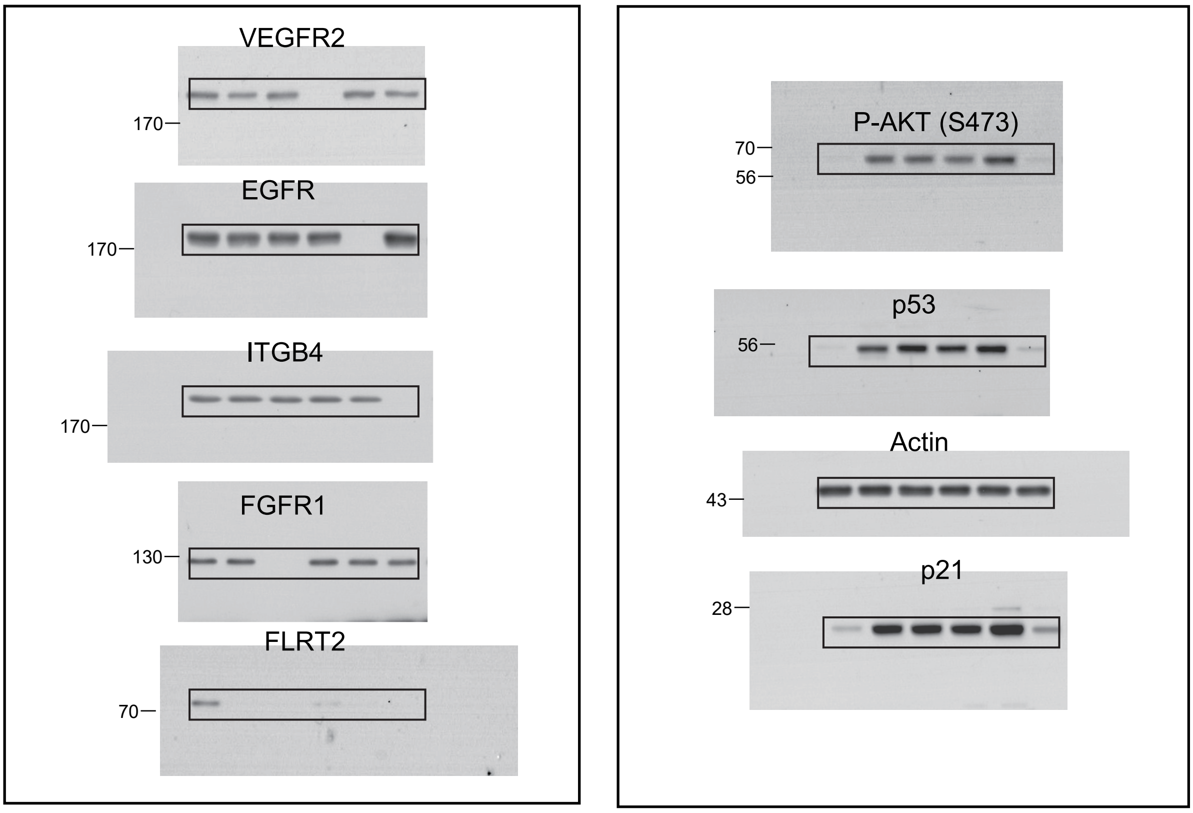

Fig. 3B

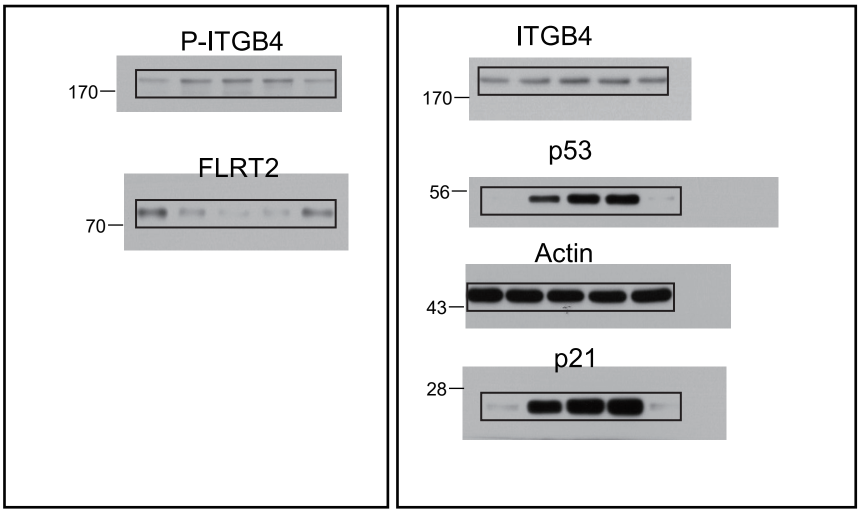

Full unedited gel for Figure 3.

Fig. 3D

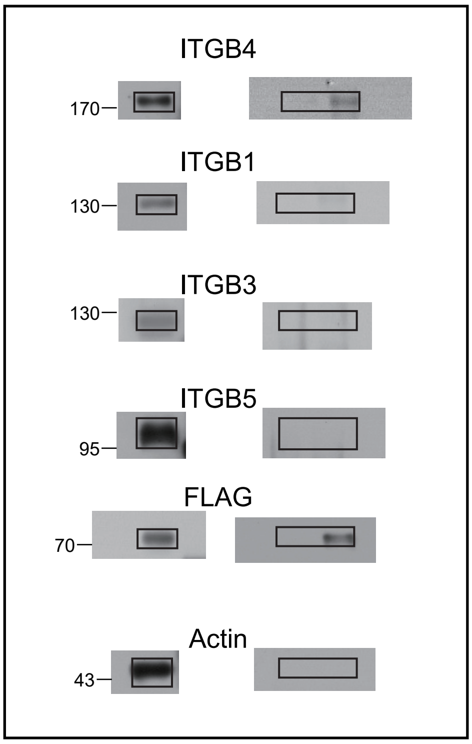

Fig. 3G

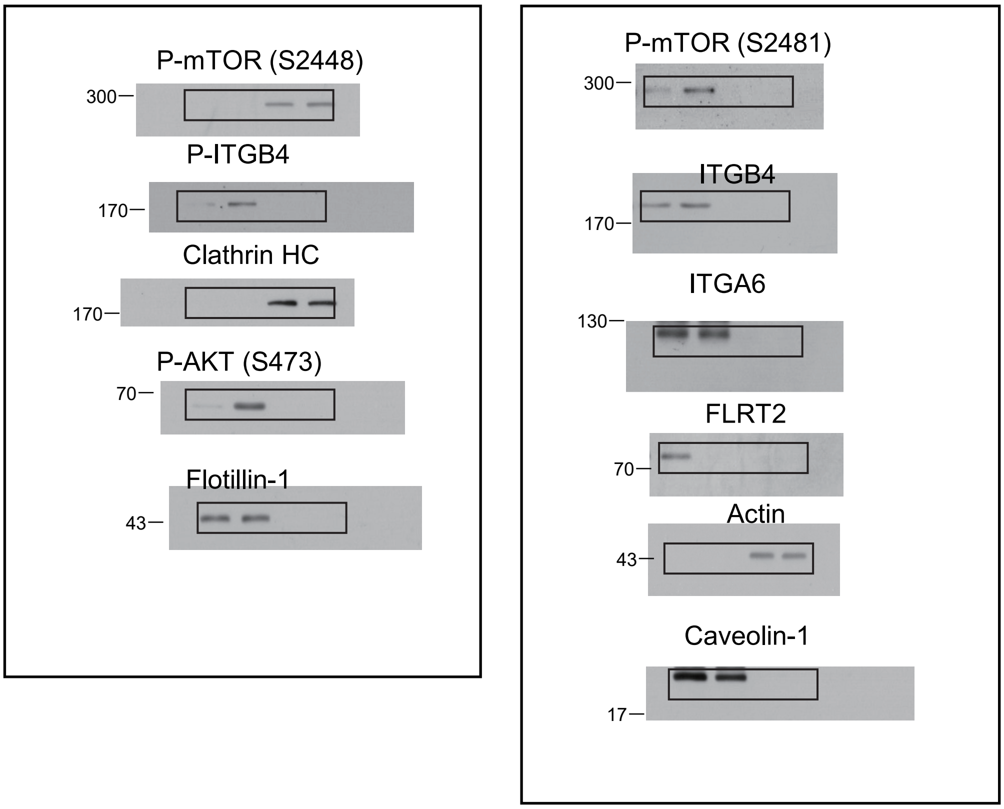

Full unedited gel for Figure 4.

Fig. 4A

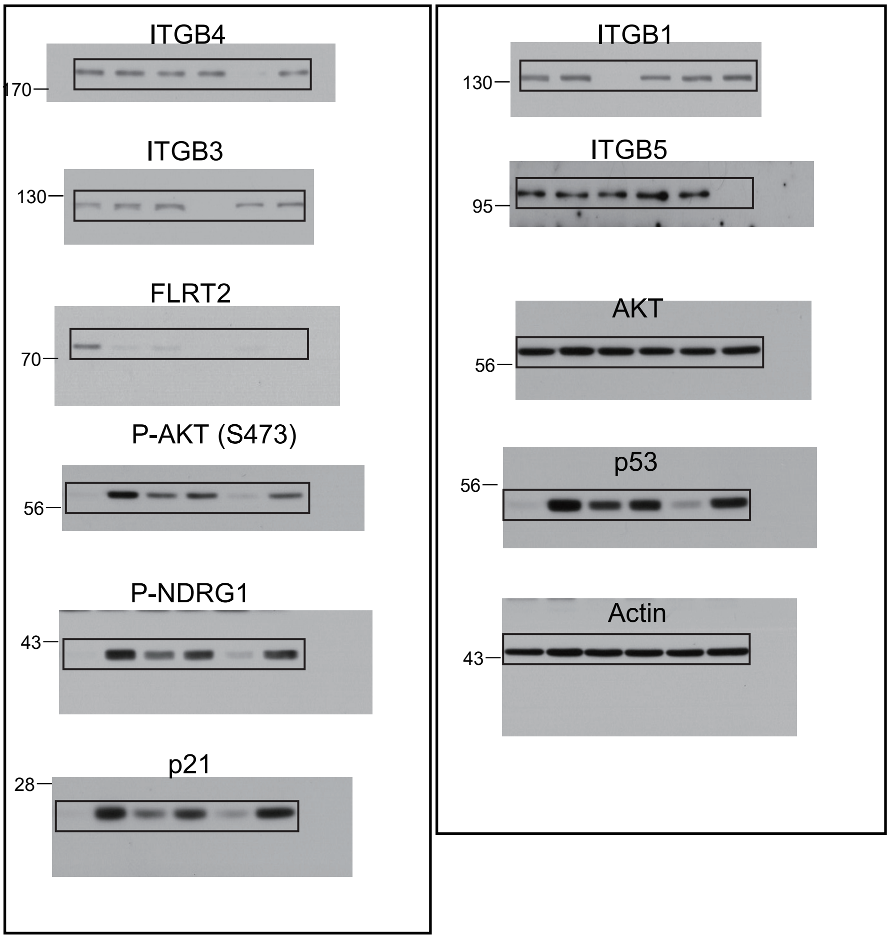

Fig. 4D

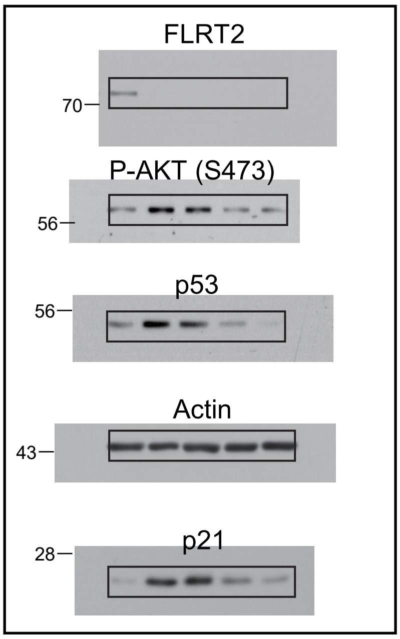

Full unedited gel for Figure 4.

Fig. 4F

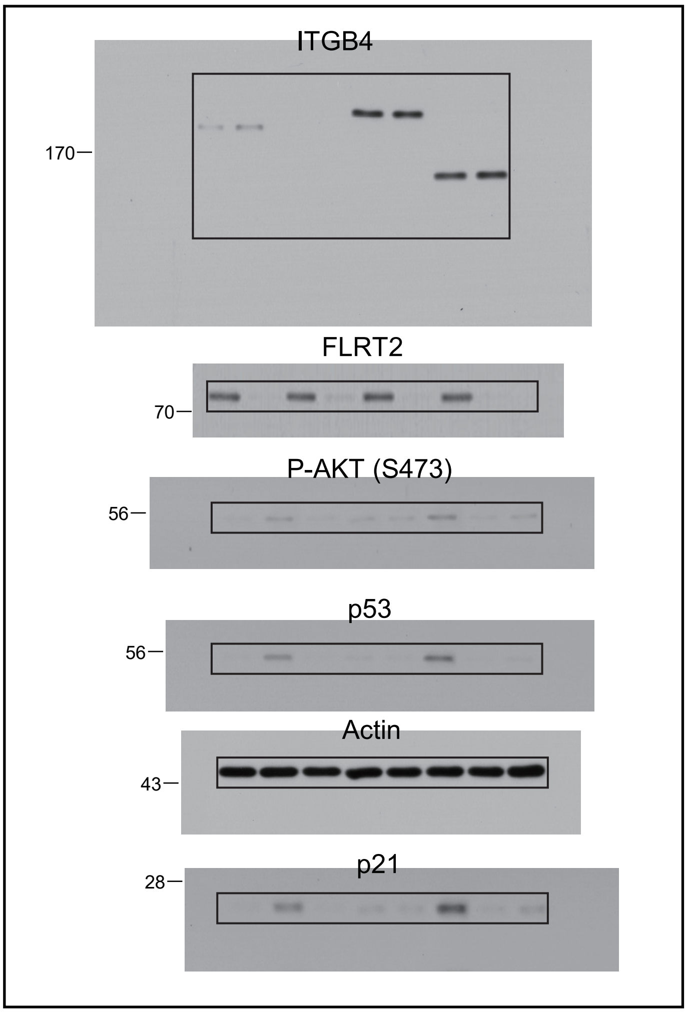

Fig. 4G

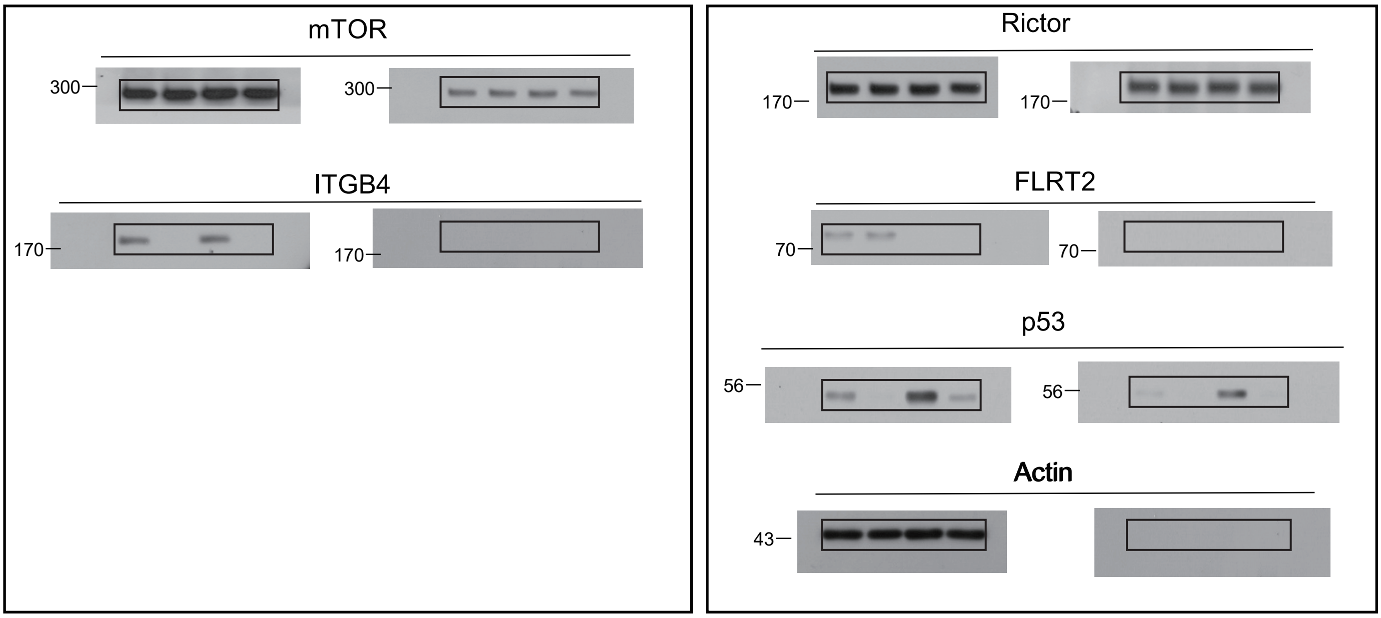

Full unedited gel for Figure 5.

Fig. 5A

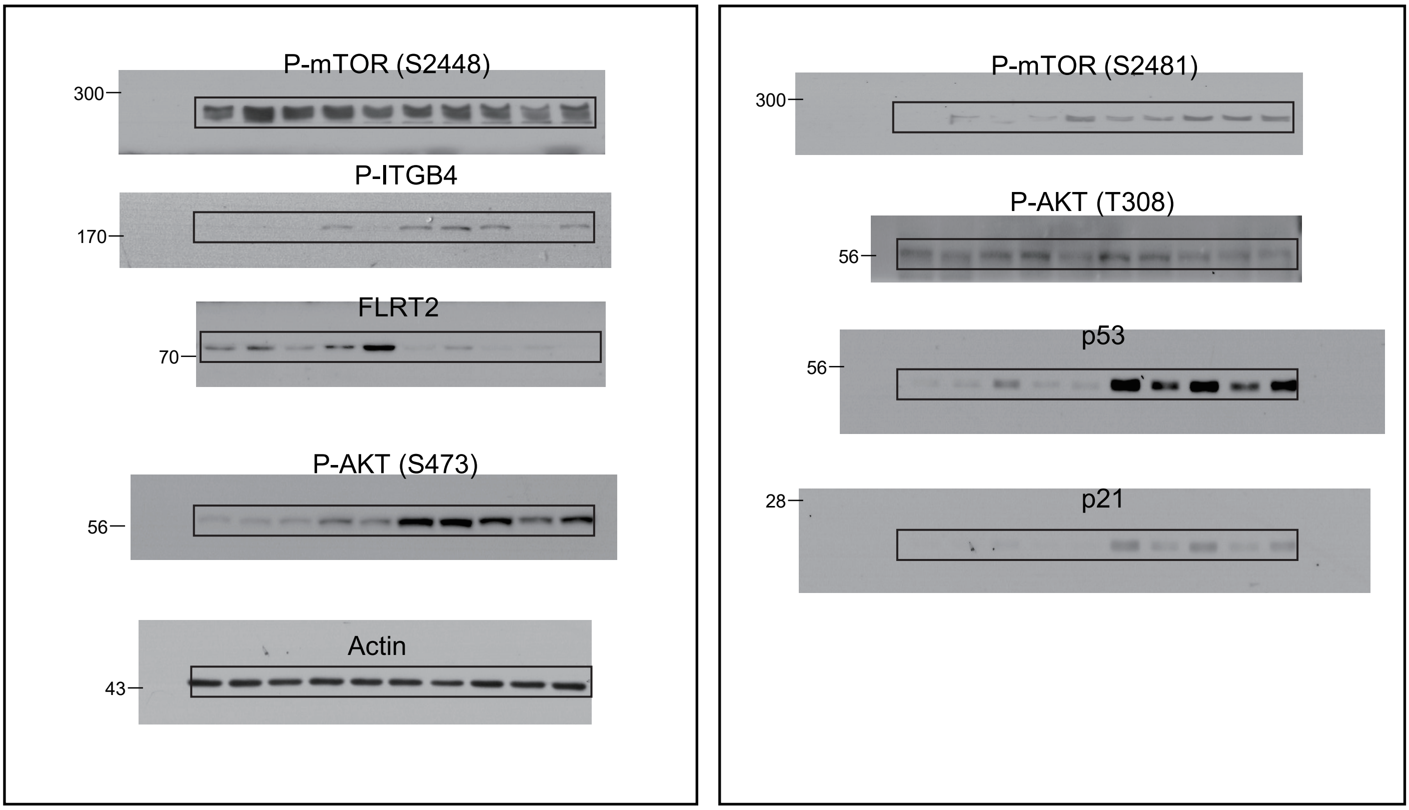

Full unedited gel for Figure 6.

Fig. 6A

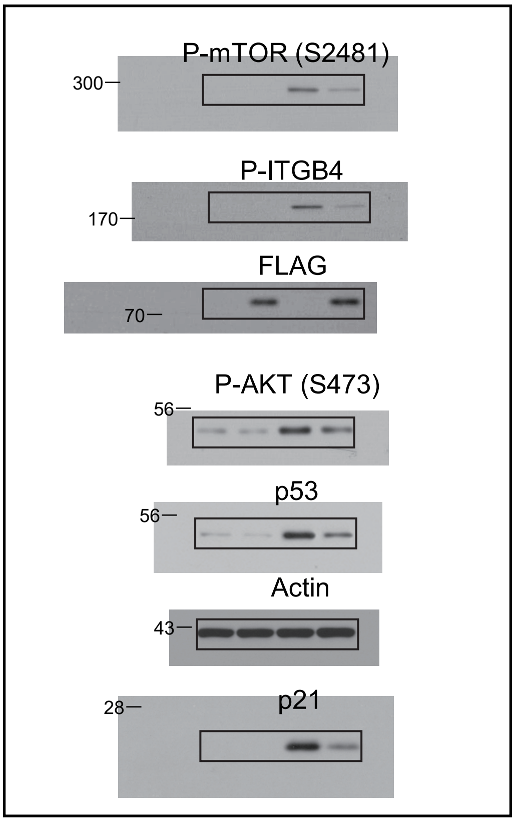

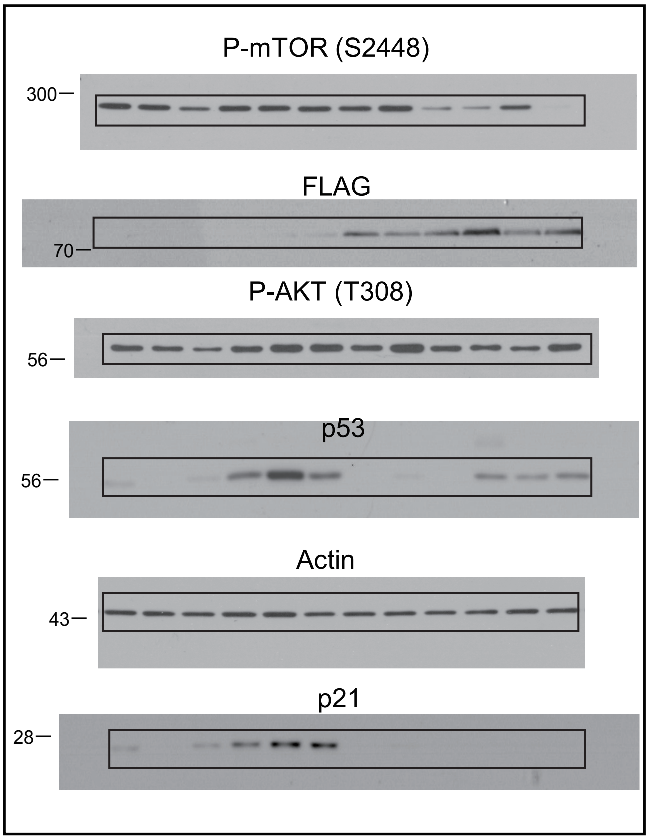

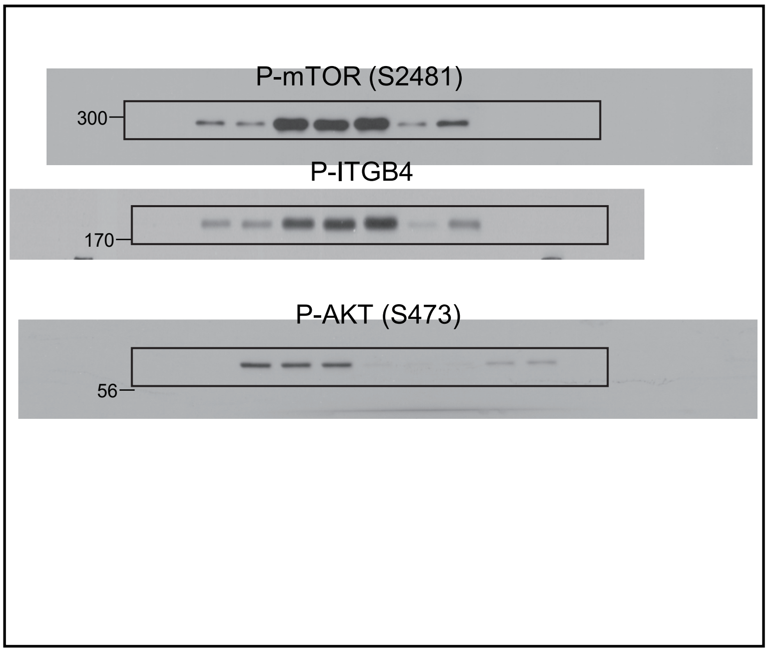

Full unedited gel for Supplementary Figure 5.

Supplementary Figure 5A

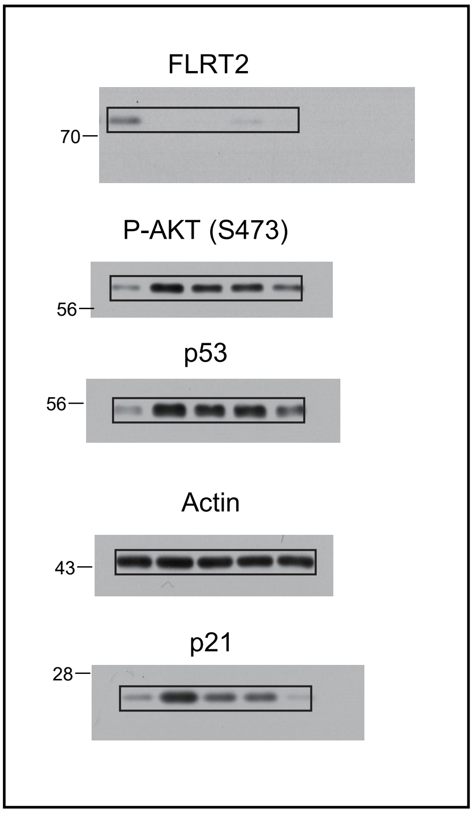

Full unedited gel for Supplementary Figure 6.

Supplementary Figure 6B

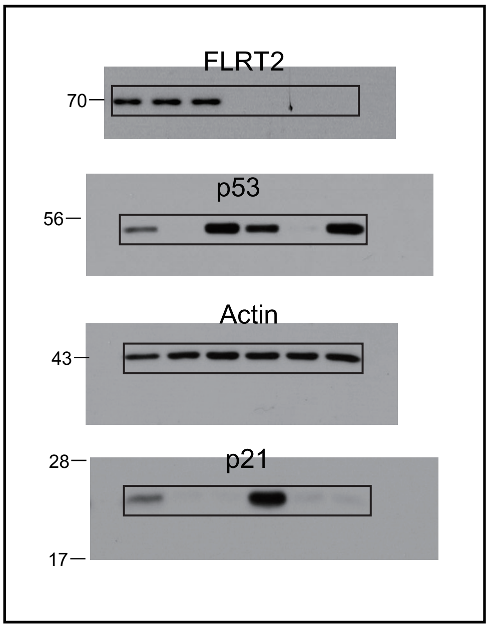

Full unedited gel for Supplementary Figure 7.

Supplementary Figure 7A

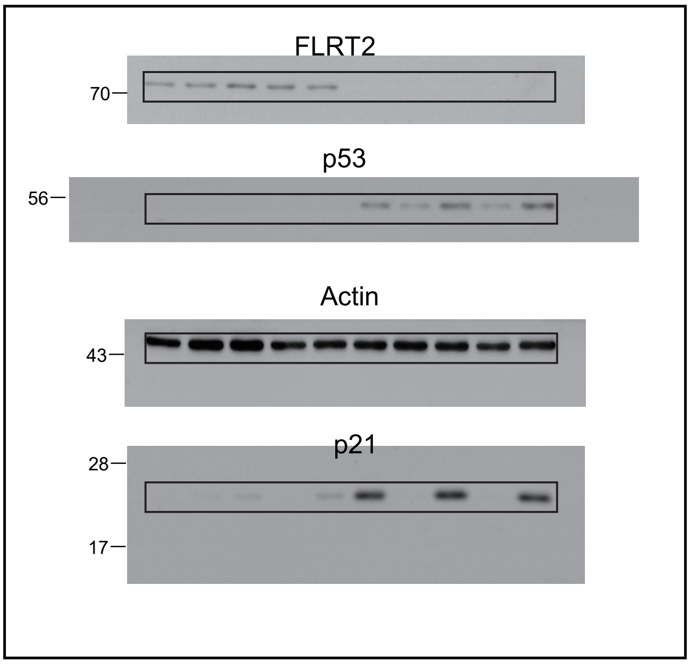

Full unedited gel for Supplementary Figure 9.

Supplementary Figure 9B

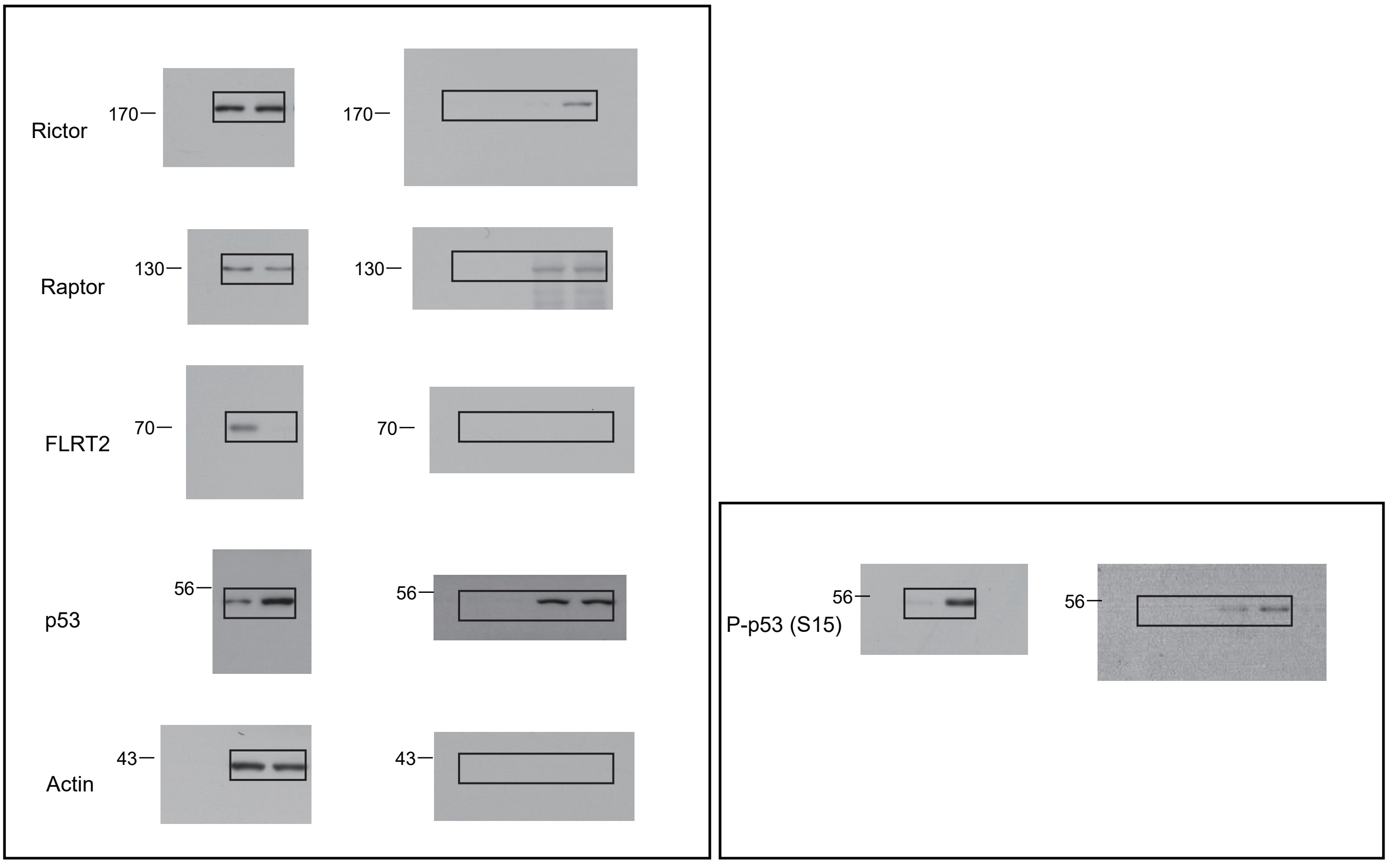

Full unedited gel for Supplementary Figure 10

Supplementary Figure 10C

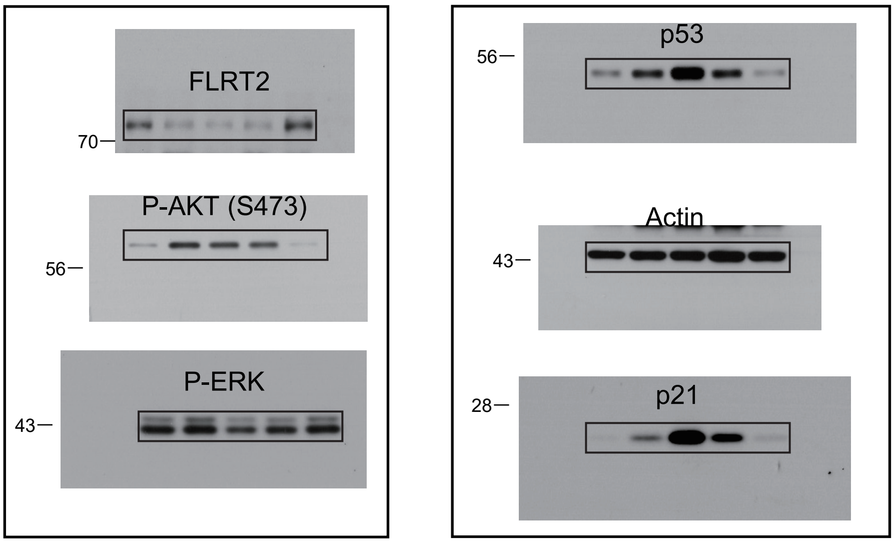

Supplementary Figure 10D

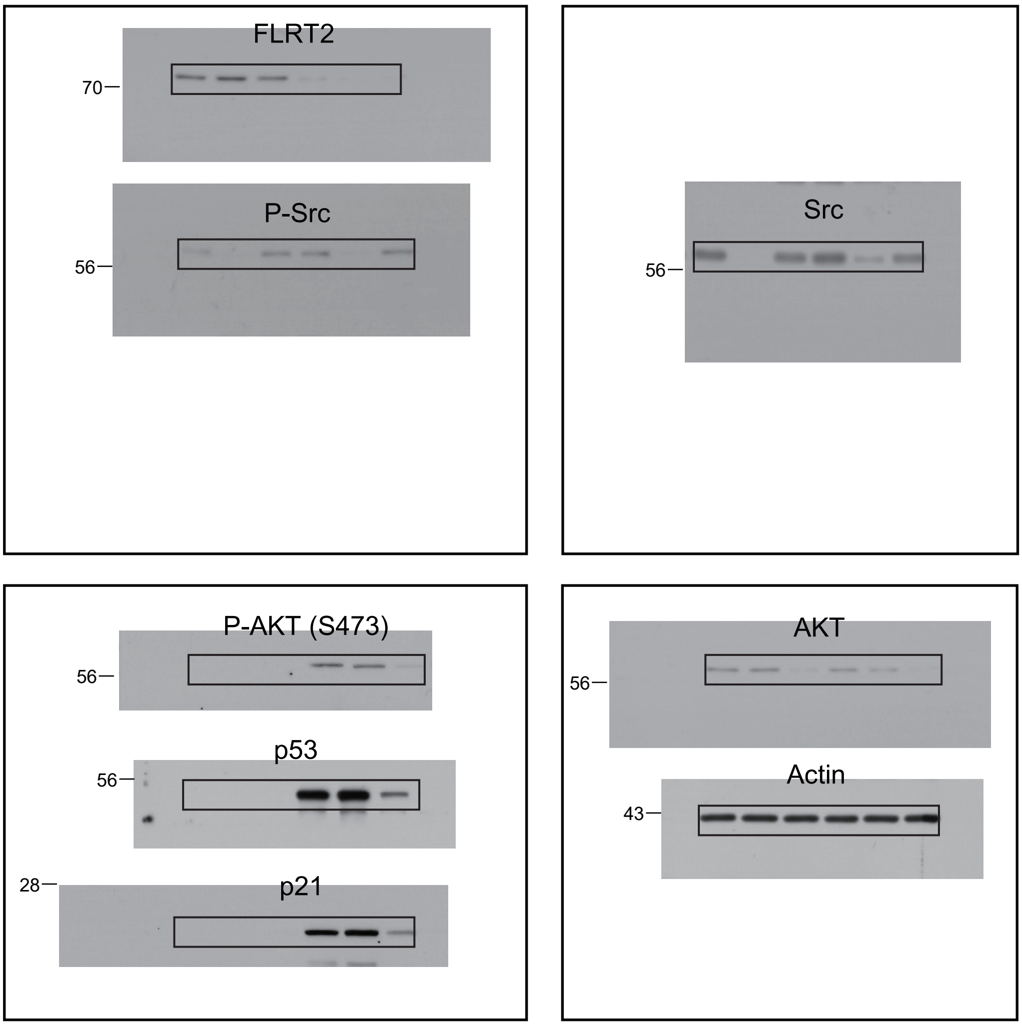

Full unedited gel for Supplementary Figure 13.

Supplementary Figure 13A

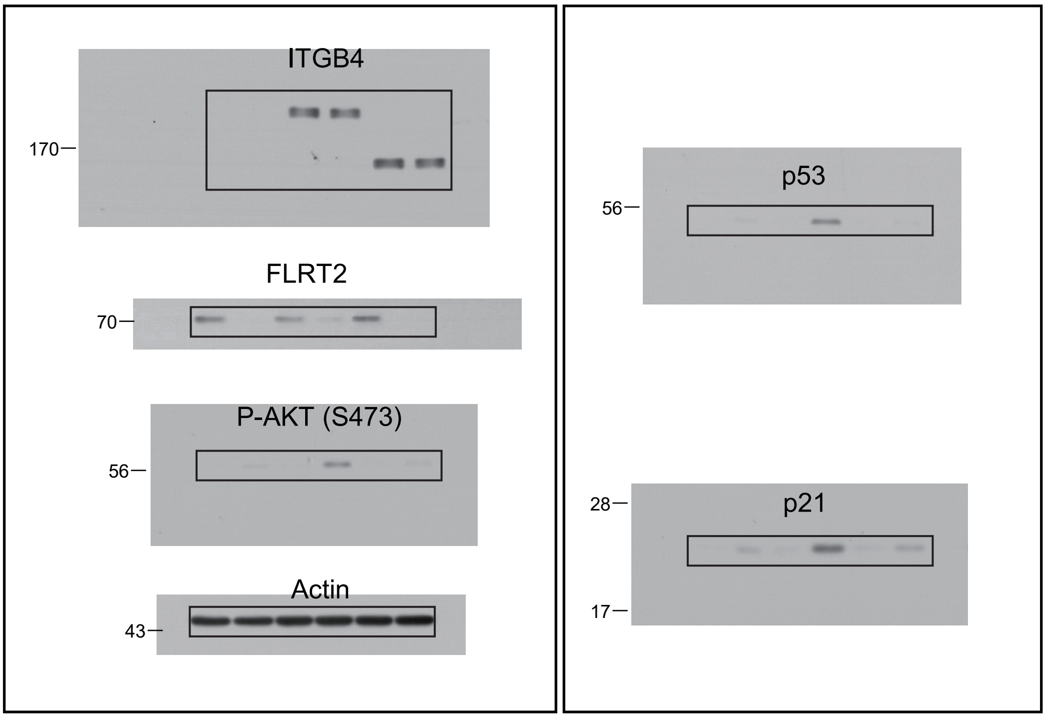

Full unedited gel for Supplementary Figure 15.

Supplementary Figure 15B

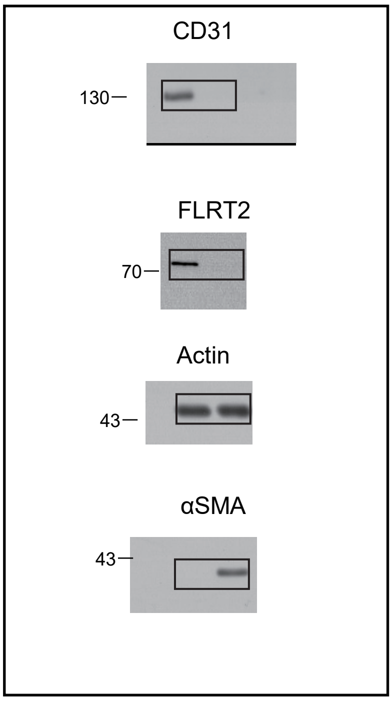

Full unedited gel for Supplementary Figure 16.

Supplementary Figure 16A

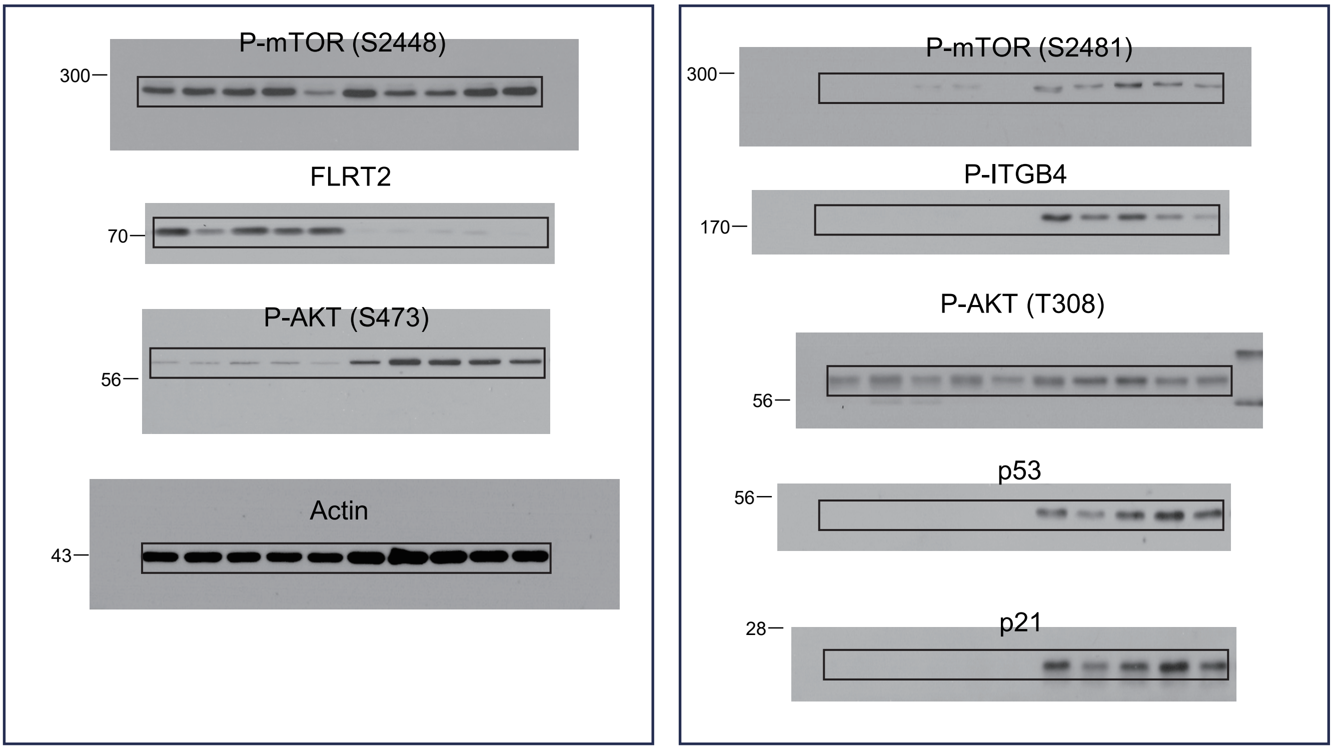

Supplement: Unedited blot and gel images [file jciinsight-9-172678-s047.pdf]
